# Optimizing Choice and Timing of Behavioral Outcome Tests After Repetitive Mild Traumatic Brain Injury: A Machine Learning-Based Approach on Multiple Pre-Clinical Experiments

**DOI:** 10.1089/neu.2022.0486

**Published:** 2023-08-16

**Authors:** Philipp Lassarén, Grace Conley, Masen L. Boucher, Ashley N. Conley, Nicholas J. Morriss, Jianhua Qiu, Rebekah C. Mannix, Eric Peter Thelin

**Affiliations:** ^1^ Department of Clinical Neuroscience, Karolinska Institutet, Stockholm, Sweden.; ^2^ Division of Emergency Medicine, Boston Children's Hospital, Boston, Massachusetts, USA.; ^3^ School of Medicine, Boston University, Boston, Massachusetts, USA.; ^4^ Duke University School of Medicine, Duke University Medical Center, Durham, North Carolina, USA.; ^5^ Harvard Medical School, Boston, Massachusetts, USA.; ^6^ Department of Neurology, Karolinska University Hospital, Stockholm, Sweden.

**Keywords:** animal studies, behavioral outcomes, Morris water maze, repetitive mild traumatic brain injury

## Abstract

Repetitive mild traumatic brain injury (rmTBI) is a potentially debilitating condition with long-term sequelae. Animal models are used to study rmTBI in a controlled environment, but there is currently no established standard battery of behavioral tests used. Primarily, we aimed to identify the best combination and timing of behavioral tests to distinguish injured from uninjured animals in rmTBI studies, and secondarily, to determine whether combinations of independent experiments have better behavioral outcome prediction accuracy than individual experiments. Data from 1203 mice from 58 rmTBI experiments, some of which have already been published, were used. In total, 11 types of behavioral tests were measured by 37 parameters at 13 time points during the first 6 months after injury. Univariate regression analyses were used to identify optimal combinations of behavioral tests and whether the inclusion of multiple heterogenous experiments improved accuracy. *k*-means clustering was used to determine whether a combination of multiple tests could distinguish mice with rmTBI from uninjured mice. We found that a combination of behavioral tests outperformed individual tests alone when distinguishing animals with rmTBI from uninjured animals. The best timing for most individual behavioral tests was 3–4 months after first injury. Overall, Morris water maze (MWM; hidden and probe frequency) was the behavioral test with the best capability of detecting injury effects (area under the curve [AUC] = 0.98). Combinations of open field tests and elevated plus mazes also performed well (AUC = 0.92), as did the forced swim test alone (AUC = 0.90). In summary, multiple heterogeneous experiments tended to predict outcome better than individual experiments, and MWM 3–4 months after injury was the optimal test, also several combinations also performed well. In order to design future pre-clinical rmTBI trials, we have included an interactive application available online utilizing the data from the study via the Supplementary URL.

## Introduction

Repetitive mild traumatic brain injury (rmTBI) is caused by relatively mild injuries in a brain that has not yet recovered from a previous mTBI.^1,2^ During the early phases after TBI, when there are numerous physiological deregulations, the brain is particularly vulnerable to new injuries, causing rmTBIs to cumulate in severity, either independently or synergistically.^1,2^ It is believed that this is why rmTBI resembles severe, rather than mild, TBI with regard to long-term outcomes.^2^ Notably, chronic traumatic encephalopathy (CTE), a neurodegenerative disease that can lead to symptoms like dementia and parkinsonism, is specifically associated with rmTBI and less with other classes of TBI.^1^ In addition to military training and combat, rmTBI is common in many sports-related injuries, and therefore impacts children and adolescents to an disproportionate degree.^1^ Thus, rmTBI may result in many years of disability, including physical, cognitive, and behavioral sequelae.^3^

Because rmTBI may lead to psychological and motor sequelae, animal tests of behavioral outcomes can be used to model these functional outcomes.^4^ Commonly, animal studies measure a set of behavioral outcomes that are considered to be analogous to those used in clinical settings, often deployed to quantify a treatment effect.^5^ However, because of the complex recovery of the injured brain and difficulty in scaling animal models to human TBI, the outcomes measured may not be optimally selected.^4^ Limited experimental resources may lead to studies with either few animals but large batteries of behavioral outcomes, or many animals but few behavioral tests, thereby risking under- or overpowering experiments, respectively.^6^ Therefore, both strategies may miss the optimal behavioral assessment of an intervention, which may result in unrefined studies that utilize more animals than are necessary to achieve the scientific goal.

In a recent large review on behavioral outcomes of TBI in rodents, Shultz and coworkers^4^ presented a complex pattern of utility of more than 20 behavioral tests at different time points after rmTBI. Some tests were reported to be most efficient at detecting effects of injury within days, others within months, whereas others were most efficient at detecting effects of injury at bimodal instances.^4^ This demonstrated that many behavioral tests have indeterminate utility.^4^ Prior studies have similarly detailed heterogeneity in efficacy, relatively low specificity, limited reproducibility, and frequent translational failures as issues with behavioral testing in animal TBI models.^4^ Rodent TBI models are often required before treatments can be studied in larger animals and in humans, so it is necessary to optimize this work in order to attain refined methods and potentially reduce animal suffering.

One way to mitigate the lack of translational robustness in behavior assays after TBI is to combine assays. There have been several attempts at constructing composite scores of behavioral tests in rodents with TBI.^7,8^ Zhao and coworkers^8^ found a number of behavioral outcomes that best predicted effects of TBI. However, although they used animals with different injury severities, they did not include mice with rmTBI.^8^ In addition, all behavioral tests were performed within four weeks of injury, thereby excluding potential long-term consequences of TBI.^8^ On the other hand, Shultz and coworkers^4^ found that some behavioral tests perform best months after rmTBI, indicating that the optimal choice and timing of behavioral testing remains unknown. This is reflected by the fact that there is no established standard battery of behavioral outcomes in TBI models, resulting in a plethora of combinations used by different groups. Presumably, this is the result of both a need within the field to investigate multiple parameters, as well as a lack of understanding of the utility of different behavioral tests.

A well-designed battery of behavioral tests should be able to detect not only the effect of injury, but also the effect of treatments designed to mitigate those effects. Classical statistics can identify combinations of behavioral tests that detect injury effects using statistical inference. However, machine learning techniques can additionally provide a performance measure of the predictability of the models.^9^ Some models can be used in both paradigms; a regression model, for example, is used for inference in classical statistics and for prediction in machine learning.^9^ A prediction approach can be used to compare statistical models internally within a given study and externally with other studies, and also provides a sense of how good the models are in terms of the performance measure.

The primary aim of this study was to identify the best combination and timing of behavioral tests to distinguish injured from uninjured animals. The secondary aim was to determine whether models using combinations of multiple heterogeneous experiments (different exposures) or individual experiments (same exposure) have the best behavioral outcome prediction accuracy.

## Methods

This is a retrospective pre-clinical study. The data set used consisted of 1203 mice retrieved from 58 different experiments conducted at the Mannix-Meehan Lab at Boston Children's Hospital, Harvard Medical School, Massachusetts, USA, some of which have been previously published independently.^10–18^ All animals were C57BL male mice (The Jackson Laboratory, Bar Harbor, Maine, USA). They were housed in a temperature- and humidity-controlled room with a 12 h light–dark cycle and fed *ad libitum*. All experiments were approved by the Boston Children's Hospital Institutional Animal Care and Use Committee (IACUC) and complied with the National Institutes of Health (NIH) Guide for the Care and Use of Laboratory Animals.

### TBI weight drop model

A modified weight drop model featuring rotational acceleration, which has been described previously for a subgroup of the data set,^13^ was used in this study. All animals were anesthetized for 45 sec with 4% isoflurane in oxygen until fully unconscious. The mice were placed on a delicate tissue (Kimwipe; Kimberly-Clark, Irving, Texas, USA) and grasped by the tail. The heads of the mice were then placed underneath tubes of varying heights such that the end of the tube was centered between and slightly in front of the ears, which approximates bregma. Finally, a 54 g metal bolt was dropped through the tube to injure the TBI mice (*n* = 675), while the sham (*n* = 528) group received comparable isoflurane exposure but did not receive the injury. The impact resulted in rotational acceleration of the animal through the Kimwipe, generating various degrees of diffuse axonal injury. In the data set analyzed in this study, a variety of injury paradigms were used, based on the height of the weight drop, and number of injuries (ranging from 0 to 13, including 1) over a given time period and age at injury (Table 1). The injury severity was assessed using the time of loss of consciousness.

**Table 1.** Characteristics of the Mice

Characteristic	TBI (*n* = 675)	Sham (*n* = 528)	All (*n* = 1203)
Height of weight drop [inch]			
0	0	528	528
28	218	0	218
42	319	0	319
46	35	0	35
50	63	0	63
60	40	0	40
Number of TBI/shams over given time period			
1 TBI/sham	116	76	192
2 TBI/shams during 1 day	28	6	34
4 TBI/shams during 4 days	56	24	80
5 TBI/shams, total	373	338	711
5 TBI/shams during 5 days	301	266	567
5 TBI/shams during 5 weeks	24	24	48
5 TBI/shams during 10 weeks	24	24	48
5 TBI/shams during 5 months	24	24	48
7 TBI/shams during 9 days	102	76	178
6-13 TBI/shams during 5 days	0	8	8
Age at TBI/sham [weeks]			
5	54	47	101
8	563	445	1008
12	35	12	47
28	23	24	47
Treatment and method of delivery			
External stimulus: Flicker	30	30	60
Genetic:	26	26	52
Tau heterozygous knockout	14	14	28
Tau homozygous knockout	12	12	24
Housing	100	61	161
Enrichment	88	61	149
Single housing	12	0	12
Intraperitoneal injection	181	55	236
*Cis* P-tau	38	9	47
CRP	6	0	6
Memantine	46	19	65
IgG	33	10	43
Saline	58	17	75
Intranasal delivery	35	12	47
Anti-CD3	17	6	23
IgG	18	6	24
Oral (via water)	48	49	97
Memantine	25	24	49
Saline	23	25	48
No treatment	255	295	550

CD3, cluster of differentiation 3; CRP, C-reactive protein; IgG, immunoglobulin G; TBI/sham, either a traumatic brain injury (TBI) or a sham injury (anesthesia without TBI).

### A note on terminology: “TBI” and “sham”

In subsequent text, figures, and tables, the terms “TBI” and “sham” are used as group terms for the action of either only anesthetizing (sham) or both anesthetizing and administering a TBI (TBI) to a mouse.

### Treatments

Mice were either exposed to a treatment (*n* = 463), to vehicle control (*n* = 190), or to no treatment (*n* = 550). No mouse was exposed to multiple treatments (Table 1). An overview of the specifics of the treatments is shown in Table 2; please see the Supplementary text for more details.

**Table 2.** Overview of the Treatments

Modality	Treatment	Details	Previous publications of this data
External stimulus	Flicker	A concurrent flash of light and sound at 40 interactions per second, theorized to restore the natural 40 Hz firing rate of γ-aminobutyric acid (GABA)-ergic neurons.^19^	—
Genetic	Tau heterozygous knockout	Transgenic mice lacked alleles of genes responsible for tau protein production. Excessive tau aggregation following injury is linked to behavioral deficits.^20^	—
	Tau homozygous knockout		
Housing	Enrichment	The Marlau™ cage has running wheels, a climbing ladder, a slide tunnel, and mazes. This promotes cognitive activity.^15^	^15^
	Single housing	Individual housing reduces cognitive activity.^21^ Other mice were placed in cages for five.	—
Intraperitoneal injection	*Cis* P-tau	*Cis* P-tau antibodies inhibit apoptosis by preventing *cis* P-tau from disrupting mitochondrial transport and axonal microtubule networks.^12^	^12,16^
	CRP	Injected monomeric C-reactive protein (CRP) promotes dementia after ischemia in mice, which is theorized to be applicable to experimental traumatic brain injury (TBI).^22^	—
	Memantine	Memantine targets *N*-methyl-d-aspartate (NMDA) receptor-mediated glutamatergic toxicity, mitigating repetitive mild (rm)TBI-induced neurological deficits.^10^	^10,11^
	IgG	Vehicle control for the *cis* P-tau antibodies. IgG = immunoglobulin G.	^12,16^
	Saline	Vehicle control for the other smaller molecules.	^10,11^
Intranasal delivery	Anti-CD3	An antibody that binds to cluster of differentiation (CD)3, which is partially responsible for T cell activation. It is theorized to mitigate a hyperactive immune response to TBI.	—
	IgG	Vehicle control for the anti-CD3 antibodies.	—
Oral (via water)	Memantine	Similar hypothesized effect as that given intraperitoneally.	—
	Saline	Vehicle control for the other smaller molecules.	—
No treatment	—	Mice naïve to any treatment or vehicle control.	—

### Behavioral outcomes: Distribution and fraction of available behavioral outcomes

**FIG. 1.**
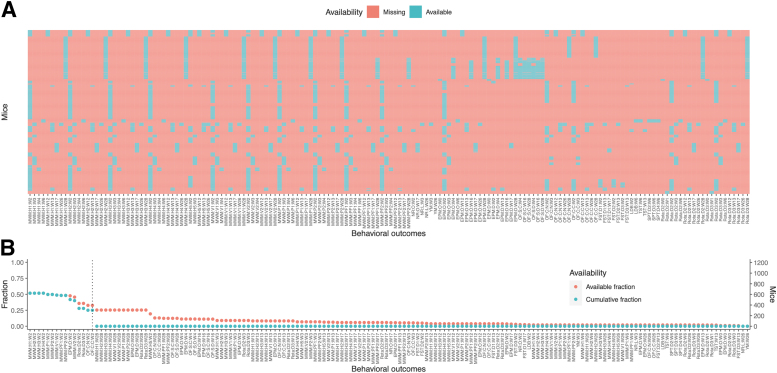
Distribution of available behavioral outcome data. **(A)** Tile plot of missing and available data. Each behavioral outcome measured at a particular time point for every mouse was plotted as “missing” or “available,” depending on whether the behavioral outcome had been tested on the mouse. The majority of tiles were missing, indicating that the data were sparse. No behavioral outcome was tested on all mice, and no mouse was tested for all behavioral outcomes. Some mice, but not all, were tested for certain outcomes at multiple time points. **(B)** Point plot of available fractions, sorted in decreasing order. For each behavioral outcome, the number of mice and the fraction of mice tested was plotted (“available fraction”). After they were ordered in decreasing order, the number of mice and fraction of mice tested for the cumulative set of behavioral outcomes up until the particular outcome were plotted (“cumulative fraction”). The behavioral outcomes with highest fractions were tested on approximately half of the mice, whereas most outcomes were tested on <10% of mice. Only 15 behavioral outcomes had a cumulative fraction >0 (vertical dashed line). EPM:D:W[…], elevated plus maze: decision: week […]; EPM:O:W[…], elevated plus maze: open arm: week […]; FST:D[…]:W[…], forced swim test: day […]: week […]; LDB:W[…], light-dark box: week […]; MWM:H[…]:W[…], Morris water maze: hidden trial […]: week […]; MWM:P[…]:W[…], Morris water maze: probe trial […]: week […]; MWM:PF[…]:W[…], Morris water maze: probe frequency trial […]: week […]; MWM:V[…]:W[…], Morris water maze: visual trial […]: week […]; NR:L:W[…], novel recognition: location: week […]; NR:O:W[…], novel recognition: object: week […]; OF:C:C:W[…], open field test: circle: center: week […]; OF:C:N:W[…], open field test: circle: neutral: week […]; OF:S:C:W[…], open field test: square: center: week […]; OF:S:D:W[…], open field test: square: distance: week […]; Rota:D[…]:W[…], rotarod: day […]: week […]; SPT:D[…]:W[…], sucrose preference test: day […]: week […]; TST:W[…], tail suspension test: week […]; YM:W[…], Y-maze: week[…].

The distribution of available data of distinct behavioral outcomes among all mice is presented in Table 3 and Figure 1A, showing great sparsity of the measured variables. Notably, no mouse was exposed to all behavioral tests, and no behavioral test was measured in all mice. The fraction of available data for each behavioral outcome, as well as the cumulative fraction, defined as the fraction of mice that were tested both for the specified behavioral outcome and for all outcomes with higher availability, is presented in Figure 1B. The combination with most variables (15 behavioral outcomes; 180 TBI and 121 sham mice) was subsequently used when complete-case data were required.

**Table 3.** Number and Timing of Measurements of the Behavioral Outcomes

	Behavioral outcome		Timing [weeks]*^a^*											Total
		0	1	2	3	4	6	12	13	16	17	26	28	
Severity of injury	Loss of consciousness	5265	0	0	0	0	0	0	0	0	0	0	0	5265
	TBI/sham 1	1146	0	0	0	0	0	0	0	0	0	0	0	1146
	TBI/sham 2	976	0	0	0	0	0	0	0	0	0	0	0	976
	TBI/sham 3	957	0	0	0	0	0	0	0	0	0	0	0	957
	TBI/sham 4	957	0	0	0	0	0	0	0	0	0	0	0	957
	TBI/sham 5	873	0	0	0	0	0	0	0	0	0	0	0	873
	TBI/sham 6	178	0	0	0	0	0	0	0	0	0	0	0	178
	TBI/sham 7	178	0	0	0	0	0	0	0	0	0	0	0	178
Cognitive	Morris water maze	0	0	6211	1038	250	144	517	910	0	700	144	2738	12,652
	Hidden trial 1	0	0	622	108	25	24	47	100	0	70	24	305	1325
	Hidden trial 2	0	0	622	108	25	24	47	100	0	70	24	305	1325
	Hidden trial 3	0	0	622	108	25	24	47	100	0	70	24	305	1325
	Hidden trial 4	0	0	622	83	25	24	47	100	0	70	24	305	1300
	Hidden trial 5	0	0	233	83	0	0	47	77	0	0	24	0	464
	Visual trial 1	0	0	586	108	25	0	47	100	0	70	24	305	1265
	Visual trial 2	0	0	548	83	25	0	47	76	0	70	0	305	1154
	Probe trial 1	0	0	598	108	25	24	47	76	0	70	0	305	1253
	Probe trial 2	0	0	598	83	25	0	47	53	0	70	0	305	1181
	Probe frequency trial 1	0	0	580	83	25	24	47	64	0	70	0	149	1042
	Probe frequency trial 2	0	0	580	83	25	0	47	64	0	70	0	149	1018
	Novel recognition	0	0	31	24	0	0	0	0	0	67	0	0	122
	Object	0	0	31	0	0	0	0	0	0	67	0	0	98
	Location	0	0	0	24	0	0	0	0	0	0	0	0	24
Anxiety/depression	Elevated plus maze	24	0	617	108	272	24	47	123	268	107	24	610	2224
	Open arm	12	0	573	108	136	24	47	100	134	67	24	305	1530
	Closed arm^b^	12	0	573	108	136	24	47	100	134	67	24	305	1530
	Decision	12	0	44	0	136	0	0	23	134	40	0	305	694
	Open field test	0	0	786	128	272	0	94	200	268	0	48	610	2406
	Square	0	0	0	0	272	0	0	0	268	0	0	298	838
	Center	0	0	0	0	136	0	0	0	134	0	0	149	419
	Wall^b^	0	0	0	0	136	0	0	0	134	0	0	149	419
	Distance	0	0	0	0	136	0	0	0	134	0	0	149	419
	Circle	0	0	786	128	0	0	94	200	0	0	48	312	1568
	Center	0	0	393	64	0	0	47	100	0	0	24	156	784
	Neutral	0	0	393	64	0	0	47	100	0	0	24	156	784
	Wall^b^	0	0	393	64	0	0	47	100	0	0	24	156	784
	Forced swim test	0	0	156	0	0	0	0	81	0	30	0	0	267
	Day 1	0	0	60	0	0	0	0	47	0	30	0	0	137
	Day 2	0	0	60	0	0	0	0	24	0	0	0	0	84
	Day 3	0	0	36	0	0	0	0	10	0	0	0	0	46
	Light-dark box	0	0	27	0	0	24	0	0	0	0	0	0	51
	Tail suspension test	0	0	0	0	0	24	0	21	0	0	0	0	45
	Sucrose preference test	0	0	0	0	0	72	0	0	0	0	0	0	72
	Day 2	0	0	0	0	0	24	0	0	0	0	0	0	24
	Day 3	0	0	0	0	0	24	0	0	0	0	0	0	24
	Day 4	0	0	0	0	0	24	0	0	0	0	0	0	24
Motor	Rotarod	24	46	860	216	0	48	94	200	0	140	48	610	2,286
	Day 2	12	23	430	108	0	24	47	100	0	70	24	305	1,143
	Day 3	12	23	430	108	0	24	47	100	0	70	24	305	1,143
	Total	5313	46	8688	1539	794	336	752	1535	536	1044	264	4568	25,415

^a^ Timing of behavioral testing is approximate. For week 0, loss of consciousness was recorded directly after injury, whereas other behavioral outcomes were recorded later during the week.

^b^ Redundant variables, with no degree of freedom.

### Statistical analysis

All analyses were performed in R, version 4.0.3.^23^

Principal component analysis (PCA). In order to visualize several behavioral outcomes simultaneously, PCA was used. PCA can reduce the number of dimensions (i.e., behavioral outcomes) to a smaller set of dimensions that account for a large fraction of explained variance. The package FactoMineR^24^ was used for calculations and plotting. All variables were scaled to unit variance, and outliers more than five standard deviations from the mean were omitted. Because PCA requires complete-case data, all analyses were performed on the first 15 behavioral outcomes in Figure 1B. First, PCA was used to visualize differences in experiments by plotting mice with no TBI and no treatment, and then coloring the mice in the new dimensions after either their experiment or total period of administered anesthesia. Ellipses of one standard deviation (68%) confidence intervals were drawn for all color groups as orientation. This could show whether inter-experiment variability could be attributed to differences in number of anesthetic events. Second, PCA was used to visualize the information content of behavioral tests. This was done by first calculating the principal components of all mice, and then plotting the correlations between the behavioral outcomes and the principal components in a heatmap. The package pheatmap^25^ was used for visualization. Dendrograms were calculated using complete-linkage clustering. In order to visualize the inter-relation of behavioral tests and their information content, behavioral outcomes were categorized according to whether higher values represent good or bad outcomes as well as whether the test group was anxiety/depression, cognitive deficit, or motor deficit.

#### Logistical model

In order to determine how well behavioral tests can discriminate between injured and uninjured animals, logistical regression was used as a supervised classifier. Because the accuracy of classification depends on a threshold value, the area under the receiver operating characteristic curve (AUC) was used as the performance measure because of its independence of the threshold.^9^ A performance measure is a quantification of the ability of a model to predict a response correctly.^9^ The AUC is an aggregated measure of the sensitivity and specificity for all thresholds.^9^ Higher AUC indicates that the model predicts injury well.^9^ The range of AUC is from 0 to 1, where AUC = 0.5 when the model is equivalent to random chance.^9^ Bootstrapping with 25 iterations was used to generate multiple data sets, from which the mean AUC and its standard deviation were recorded. This maximized use of the limited data points, while still allowing for validation across the iterations. For completeness, the data were also run using 10-fold cross- validation, with the results displayed in the accompanying Shiny^26^ application. In order to avoid overfitting, a minimum of 10 mice per variable were required for each model to be run. To account for inter-experiment variability, all models were adjusted for number of TBI/shams. Calculations were done using the package caret.^27^

#### Best model selection algorithm

To find the best model, the following algorithm was used: All behavioral outcomes at level 3 were grouped by week of testing (Table 4). For each group of level 3 behavioral outcomes at a certain week (e.g. Morris water maze: hidden trials 1–5: week 17 [MWM:H1:W17–MWM:H5:W17]), AUC was computed for all possible combinations of behavioral tests. The best combination was recorded. If the best AUC minus one standard deviation of AUC of this group was >0.75, the best combination qualified for further analysis in step 2. Qualified combinations were labeled as the best models for level 3. All qualified models from best models for level 3 were candidates to be included at level 2. For each level 2 behavioral outcome (e.g,. all MWMs), all qualified variables from level 3 were selected from the data. Because of the high degree of missingness, where there were no mice undergoing all tests, all variables could not be included simultaneously. Therefore, the combination with the highest possible number of variables was included and labeled as the maximum model. From the maximum models, the best combinations were recorded as the best models for level 2. All qualified models from best models for level 2 were candidates to be included at level 1. The selection algorithm for best models for level 1 was analogous to that previously explained for level 2.

**Table 4.** Overview of the Behavioral Outcomes

Behavioral outcome			Details	Previous publications of this data
Level 1	Level 2	Level 3		
Cognitive	Morris water maze (MWM)	Hidden (H)	Mice were placed in a water tank with a hidden platform that they could mount to rest. Latency to find the platform without (H) and with visual (V) cues was recorded. The platform was removed and time spent (P) at the previous location of the platform or number of times passed by (PF) was recorded. Rapid location of the platform or much time spent in its previous location is associated with good spatial learning and memory.^28^	^11-18^
		Visual (V)		
		Probe (P)		
		Probe frequency (PF)		
	Novel recognition (NR)	Object (O)	Mice were allowed to become familiarized with three identical objects. One of the objects was either replaced by a novel object (O) or moved to a novel location (L). Much time spent at the novel object or location is associated with good working memory.^29^	^16^
		Location (L)		
	Y-maze (YM)		Mice were placed in the center of a Y-shaped container with three identical arms oriented at 120 degree angles. A large alternation between arms is associated with good spatial working memory.^30^	—
Anxiety/ depression	Elevated plus maze (EPM)		Mice were placed in the center of a platform with two open and two closed (walled) arms. More time spent in the open arms is associated with lower levels of anxiety.^31^	^11,12,15,17,18^
	Open field test (OF)	Square — position (SP)	Mice were placed in either circular (C) or square (S) opaque containers, facing a wall. Position (P) and distance (D) travelled were recorded. More time spent in the central areas of the open field is associated with low levels of anxiety.^32^	^11,15,18^
		Square — distance (SD)		
		Circle — position (C)		
	Forced swim test (FST)		Mice were placed in a water-filled glass container (Ø 18 cm). More time spent immobile is associated with depressant-like behavior.^33^	^15^
	Light-dark box (LDB)		Mice were placed in a dark chamber, connected to a brightly lit box. Less locomotor activity is associated with anxiety-like behavior.^34^	—
	Tail suspension test (TST)		Mice were suspended by the tail. More time spent immobile is associated with depressant-like behavior.^35^	—
	Sucrose preference test (SPT)		Mice were placed in a cage with two sipper tubes, one of which contained normal drinking water and the other with 2% sucrose added. Lower intake of sucrose solution is associated with depressant-like behavior.^36^	—
Motor	Rotarod (Rota)		Mice were trained by being placed on the rotating drum (4 rpm). Testing was performed the following days. Mice were placed on the rotating drum (4 rpm) for 10 sec to acclimate, after which the rod was accelerated by 0.1 rpm/sec. Shorter latency to fall is associated with greater neurological deficit.^37^	^11,15,16,18^

#### k-means clustering

To explore the data structure of combinations of behavioral outcomes, k-means clustering was used. Compared with the supervised learning of logistical regression, k-means clustering is an unsupervised machine learning algorithm.^9^ Therefore, it finds clusters in the data, entirely based on the behavioral outcomes (without training on injury data). The algorithm partitions the data into k non-overlapping clusters.^9^ The adjusted Rand index (ARI), which is an adjusted fraction of correct clustering, was used as the performance measure.^38^ If the clusters are more similar to the true division between injured and uninjured animals, the ARI is higher. The ARI is bounded above at 1, and an ARI of 0 occurs at random chance.^38^ The same complete-case data set as in the aforementioned PCA was used. All combinations of behavioral outcomes included were run through the algorithm, with from 1 to 15 variables in each model. All variables were scaled to unit variance, and outliers more than five standard deviations from the mean were omitted. Calculations were done using the stats package in base R.^39^ The Euclidean metric and the algorithm of Hartigan and Wong^40^ was used. Visual analysis of the feature distribution showed generally convex distributions (Supplementary Figures).

#### Linear model

To determine whether multiple heterogeneous experiments can predict outcome better than single homogenous experiments, linear regression, a supervised learning algorithm, was used. The performance measure was the root-mean-square error (RMSE) divided by the standard deviation of the behavioral outcome data (standardized RMSE). If the model predicts outcome well, the RMSE is lower. The dependent variable chosen to investigate was MWM:H4:W2; a pilot study not shown indicated a similar pattern for most behavioral outcomes. Independent variables were loss of consciousness after the first TBI/sham, number of TBIs/sham exposures, and treatment. For each number of studies to be included (ranging from 1 to 32), 25 random combinations of studies were selected, and for each of these, bootstrapping was used with 10 iterations. Calculations were done using the package caret.^27^ All outliers more than five standard deviations from the mean were omitted prior to calculations.

### Ethical approval

All experiments were approved by the Boston Children's Hospital Institutional Animal Care and Use Committee and complied with the NIH Guide for the Care and Use of Laboratory Animals. Ethical permit has been approved under the following review numbers: 17-09-3532R, 18-07-2754R, 18-12-3851R, 19-12-4110R*, 20-04-4163R, and 20-08-4251R. The experiments presented have not primarily been used for the outlined analysis, but rather were retrospectively collected from materials that have been or will be published separately. In terms of the 3R principle^41^ – replace, refine, and reduce animal experiments – this study is a way to refine the original data by maximizing output of already recorded data. Its results might be used to refine future animal experiments.

## Results

### Inter-experiment variability was attributed to number and timing of anesthetic events

**FIG. 2.**
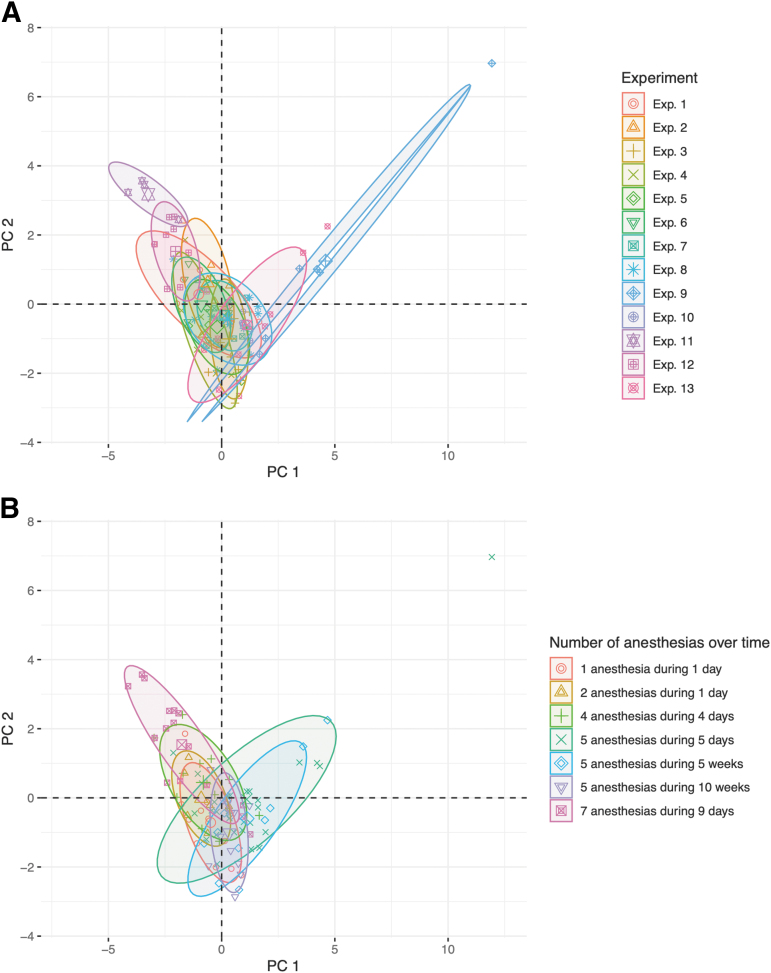
Principal component analysis (PCA) of mice with no traumatic brain injury (TBI) and no treatment. All mice with no TBI and no treatment from the 15 variables with cumulative available fractions > zero (*n* = 100) were included. PCA was performed and the scores of the first two principal components (PC) were plotted. Mice were colored after **(A)** experiment or **(B)** number of anesthesias. Ellipses drawn cover a one-standard-deviation (68%) confidence interval. Mice of two experiments (11 and 12) were relatively concentrated in one area of the plot and to a large extent separated from the other mice **(A)**. The same mice had seven anesthesias, compared with the other mice that had fewer **(B)**. PC, principal component.

Inter-experiment variability was investigated using PCA on mice with no TBI or treatment (*n* = 100). Of the 13 experiments included in the PCA, two experiments (11 and 12) deviated from the others (Fig. 2A)1. However, when grouping the same data by number of anesthetic events and total period over which these were administered, mice in the outlying experiments were anesthetized seven times as opposed to the fewer anesthetizations administered to the other mice (Fig. 2B). Hence, differences in inter-experiment variability for a subset of mice with no TBI and no treatment were attributed to number and timing of anesthetic events. Whenever possible, adjustments were henceforth made based on the number of TBIs/sham procedures.

### Information contained by behavioral outcomes was directionality and type

**FIG. 3.**
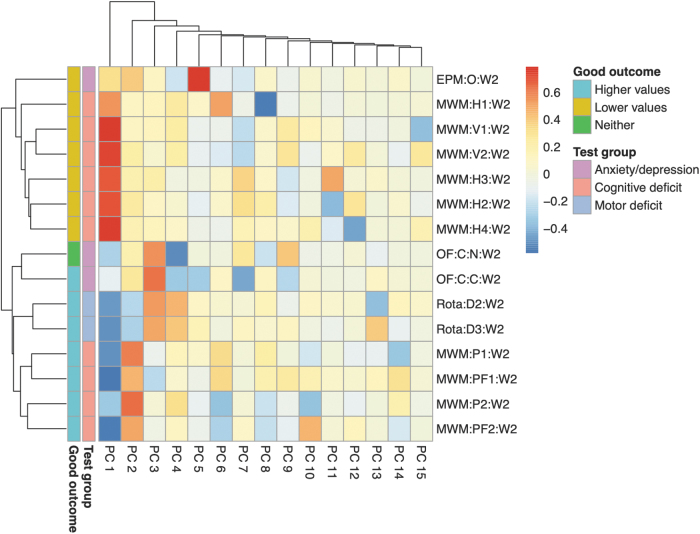
Heatmap of correlations between behavioral outcomes and their principal components. All mice from the 15 variables with cumulative available fractions > zero (*n* = 301) were included. Principal component analysis (PCA) was performed, and the correlations between the behavioral outcomes and the principal components were plotted as a heatmap. The heatmap was annotated with (1) whether higher or lower values are associated with good outcome (“Good outcome”) and (2) which level 1 group the behavioral outcomes belong to (“Test group”). The first principal component (PC 1) was most associated with “Good outcome,” whereas PCs 2, 3, and 4 were most associated with “Test group.” EPM:O:W[…], elevated plus maze: open arm: week […]; MWM:H[…]:W[…], Morris water maze: hidden trial […]: week […]; MWM:P[…]:W[…], Morris water maze: probe trial […]: week […]; MWM:PF[…]:W[…], Morris water maze: probe frequency trial […]: week […]; MWM:V[…]:W[…], Morris water maze: visual trial […]: week […]; OF:C:C:W[…], open field test: circle: center: week […]; OF:C:N:W[…], open field test: circle: neutral: week […]; PC, principal component; Rota:D[…]:W[…], rotarod: day […]: week […].

Information content of behavioral outcomes was investigated using PCA (*n* = 301, 180 TBI and 121 sham mice). Correlations of behavioral outcomes and principal components are shown in a heatmap (Fig. 3). The first principal component divided the behavioral outcomes by whether higher or lower “scores” on the behavioral test were associated with more beneficial outcomes (“directionality”). The second, third, and to some extent, fourth principal components divided the behavioral outcomes by their level 1 group (anxiety/depression, cognitive deficit, and motor deficit). Hence, the first few principal components contained essential information of the behavioral outcomes. Because directionality does not influence the performance of logistical regression, subsequent results could be interpreted in terms of behavior type.

### MWM could best distinguish injured from uninjured animals after 3–4 months

**FIG. 4.**
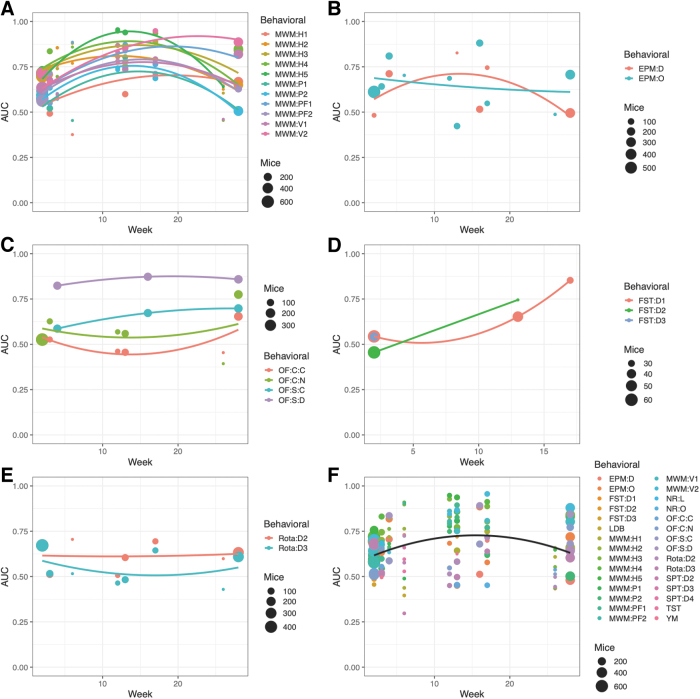
Area under the receiver operating characteristic curve (AUC) of injury prediction by each behavioral outcome using logistical regression. For each behavioral outcome at level 3, logistical regression was performed on all tested mice. Bootstrapping was used with 25 iterations, and the models were corrected for number of traumatic brain injuries (TBI)/shams. Quadratic parabolas were fitted to the data. For the Morris water maze **(A)**, most parabolas of AUC were maximized ∼3–4 months after injury. For other behavioral outcomes, maxima were not as evident, although some trend could be observed when all outcomes were combined **(F)**. **A–E** Display one level 2 behavioral outcome each. **(F)** Displays all behavioral outcomes. EPM:D:W[…], elevated plus maze: decision: week […]; EPM:O:W[…], elevated plus maze: open arm: week […]; FST:D[…]:W[…], forced swim test: day […]: week […]; LDB:W[…], light-dark box: week […]; MWM:H[…]:W[…], morris water maze: hidden trial […]: week […]; MWM:P[…]:W[…], morris water maze: probe trial […]: week […]; MWM:PF[…]:W[…], morris water maze: probe frequency trial […]: week […]; MWM:V[…]:W[…], morris water maze: visual trial […]: week […]; NR:L:W[…], novel recognition: location: week […]; NR:O:W[…], novel recognition: object: week […]; OF:C:C:W[…], open field test: circle: center: week […]; OF:C:N:W[…], open field test: circle: neutral: week […]; OF:S:C:W[…], open field test: square: center: week […]; OF:S:D:W[…], open field test: square: distance: week […]; Rota:D[…]:W[…], rotarod: day […]: week […]; SPT:D[…]:W[…], sucrose preference test: day […]: week […]; TST:W[…], tail suspension test: week […]; YM:W[…], Y-maze: week […].

**FIG. 5.**
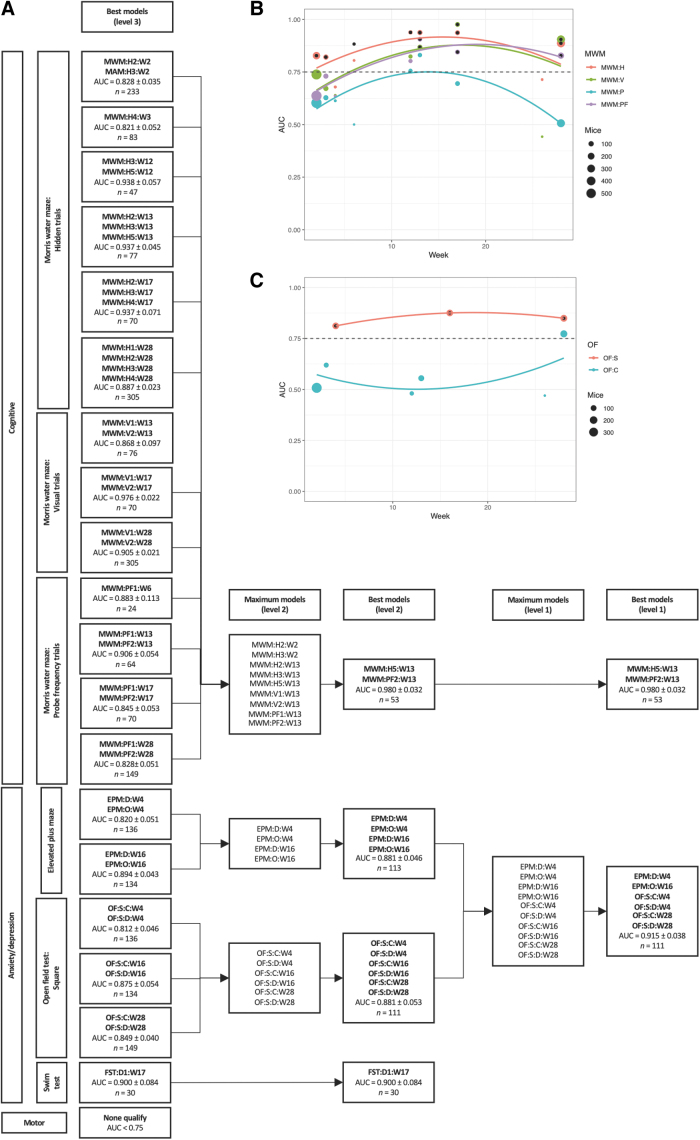
Area under the receiver operating characteristic curve (AUC) of injury prediction by combinations of behavioral outcomes using logistical regression. All behavioral tests were grouped by time after first injury. Parameters from the smallest unit of each test were grouped in level 3; for example, Morris water maze (MWM) (hidden trials 1–5), open field (OF) test (squared arena), or forced swim test (days 1–3). For every week and level 3 test, the best logistical classification model in terms of the AUC was identified. Bootstrapping was used with 25 iterations, and the models were corrected for number of traumatic brain injuries (TBI)/shams. If the best model had an AUC – standard deviation(AUC) >0.75, it qualified to be included for further analysis and was displayed in the “Best models (level 3)” column of **(A)**. All top models for every level 3 MWM and OF test are shown in **(B)** and **(C)**, respectively to illustrate examples of timing dependency. Models over the AUC threshold were included in the “Best models (level 3)” column **(A)** and denoted with a black mark in **(B)** and **(C)**. All parameters included in the models of the “Best models (level 3)” column of **(A)** were eligible to be included in a mixed analysis of all level 2 groups; for example, the MWM, OF test, or forced swim test. Because of missingness in the data, only behavioral tests contributing to a complete-case matrix with the most number of behavioral tests could be included. These are displayed in the “Maximum models (level 2)” column of **(A)**. Of these, the best model was identified analogously to level 1 and was displayed in the “Best models (level 2)” column of **(A)**. Similarly, the columns “Maximum models (level 1)” and “Best models (level 1)” of **(A)** were created, where level 1 refers to the type of test (cognitive, anxiety/depression or motor). Among the cognitive tests, a combination of MWM tests was the best, with AUC = 0.980 ± 0.032. Among the anxiety/depression tests, a combination of elevated plus maze (EPM) and OF test was the best, with AUC = 0.915 ± 0.038. No behavioral test within the motor group qualified to the initial AUC threshold. Therefore, no motor test was included in this analysis. EPM:D:W[…], elevated plus maze: decision: week […]; EPM:O:W[…], elevated plus maze: open arm: week […]; FST:D[…]:W[…], forced swim test: day […]: week […]; MWM:H[…]:W[…], Morris water maze: hidden trial […]: week […]; MWM:PF[…]:W[…], Morris water maze: probe frequency trial […]: week […]; MWM:V[…]:W[…], Morris water maze: visual trial […]: week […]; OF:S:C:W[…], open field test: square: center: week […]; OF:S:D:W[…], open field test: square: distance: week […].

The performance of distinguishing injured from uninjured animals was investigated using logistical regression. The highest AUC was observed for MWM when measured ∼3–4 months after first TBI/sham (Fig. 4A). Total sample sizes are shown as bullet points in the subfigures 4A–E and 5B and C; exact values and proportions of TBI and sham mice can be found in the online application that can be accessed via the Supplementary URL. Global maxima were less apparent for the other behavioral tests shown (Fig. 4B–E). At the respective maxima, MWM outperformed all other tests in terms of AUC. The compilation of all tests (Fig. 4F) also pointed toward an average maximum AUC after ∼3–4 months from first TBI/sham, but it was heavily weighted by the abundance of data for MWM. When grouped by level 3 tests (e.g., MWM hidden trials, MWM:H), similar time trends were observed (Fig. 5B and C).

### MWM:H5:W13 and MWM:PF2:W13 could together best distinguish injured from uninjured animals

Overall, the best combination of behavioral tests was found to be MWM hidden trial 5 (MWM:H5:W13) and MWM probe frequency trial 2 (MWM:PF2:W13) during week 13, yielding an AUC of 0.980 ± 0.032 (Fig. 5A). Total sample sizes are displayed in Figure 5A; proportions of TBI and sham mice can be found in the online application that can be accessed via the Supplementary URL. For the best performing model, the sample size was 53 (36 TBI and 17 sham mice). Numerous MWM tests had sufficient AUC to qualify at level 3, indicating that several tests had high capability of discriminating injured from uninjured animals. However, from a total of nine MWM tests at level 2, the best combination involved only two tests (MWM:H5:W13 and MWM:PF2:W13), indicating their relative importance.

### A combination of elevated plus maze (EPM) and open field (OF) tests were second best at distinguishing injured from uninjured animals

From the anxiety/depression group, the best combination of behavioral tests was found to be a combination of two EPM (at weeks 4 and 16) and four OF tests (at weeks 4 and 28) (Fig. 5A). The sample size was 111 (56 TBI and 55 sham mice). The AUC for this combination was 0.915 ± 0.038, which was lower than that found for the combination of MWM tests previously presented, but still with a high discriminative capacity. At level 3, the forced swim test (FST) (at week 17) performed well with an AUC of 0.900 ± 0.084 (*n* = 30; 15 TBI and 15 sham mice), but could not be included in the maximum model at level 1, because the other behavioral outcomes in the anxiety/depression group were not tested on the same animals. No motor test qualified at level 3 because of low performance of the rotarod test (Fig. 4E).

### An interactive online application allows the reader to try their own combination

In order to allow the reader to test their own preferred combinations of behavioral tests on this dataset, an interactive online application has been created in the R package “Shiny.”^26^ In addition to the test results using bootstrapping, the application includes results from cross-validation. It is available via the Supplementary URL.

### Multiple behavioral tests created clusters around injured and uninjured animals

**FIG. 6.**
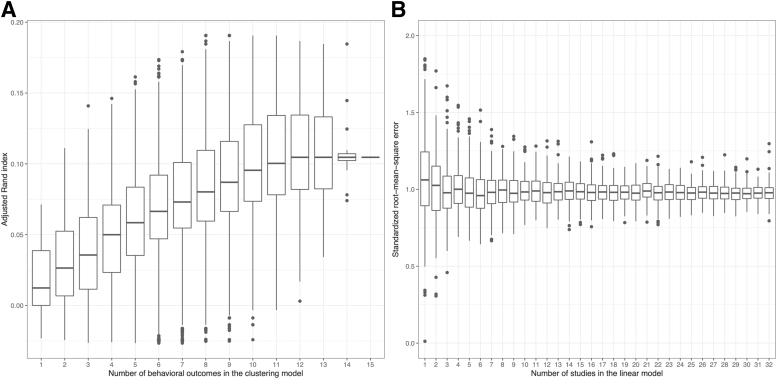
Performance when cumulating behavioral outcomes or studies in models. **(A)** All mice from the 15 variables with cumulative available fractions > zero (*n* = 301) were included. Using the partitions created from *k*-means clustering on all combinations of 1–15 behavioral outcomes, the adjusted Rand index (ARI) was computed. Although the highest median ARI was ∼0.1, there was a relative increase in ARI with more behavioral outcomes. At six behavioral outcomes, the central quartiles do not overlap with those at one behavioral outcome. **(B)** Studies testing the Morris water maze: hidden trial 4; week 2 (MWM:H4:W2) were included. Linear models were run to predict behavioral outcome, using number of traumatic brain injuries (TBI)/shams, treatment, and loss of consciousness after the first TBI/sham as predictors. For each number of studies to be included, 25 randomly selected study combinations were computed. Bootstrap with 10 iterations was used for each of these combinations. The root-mean-square error (RMSE), divided by the standard deviation, was used as the performance measure. The median standardized RMSE decreased with more behavioral outcomes, but considering the overlapping central quartiles, this should be interpreted with caution.

The ARI improved with more behavioral tests that were included in the *k*-means clustering algorithm (Fig. 6A). The sample size was 301 (180 TBI and 121 sham mice). With six behavioral tests included in the model, the box was in its entirety higher than the box for a single behavioral test. However, the variance of the data should be interpreted with caution, as by construction, the number of possible combinations ranges from 1 (for 15 behavioral tests) to 6435 (for 7 and 8 behavioral tests). A plateau could be noticed at ∼12 behavioral outcomes and ARI ≈0.1 (at chance, ARI = 0). Hence, when used together, behavioral outcomes cumulatively structured the data in injured and uninjured groups.

### A combination of multiple heterogenous experiments tended to predict behavioral outcomes better than individual homogeneous experiments

The standardized RMSE decreased slightly for the first three studies that were included in a linear model predicting MWM:H4:W2, but plateaued for subsequently added studies (Fig. 6B). Sample size increased on average linearly from a median of 20 for 1 study to 610 for 32 studies. The standardized RMSE converged at ∼1 for the studies, corresponding to an error of one standard deviation. The boxes of the boxplots were overlapping, so the trend should be interpreted cautiously.

## Discussion

We found that a combination of two measures of the MWM at ∼3–4 months following the first injury performed best overall in discriminating injured from uninjured animals in a closed head rmTBI model. From the anxiety/depression category of behavioral tests, a combination of the OF test and EPM performed well. In addition, as a single test, the FST, had high performance. Combinations of multiple parameters of behavioral tests outperformed single tests. We found that errors tended to decrease with larger collections of heterogeneous studies, but a significant trend could not be established.

### Timing of experiments is relevant

In our analysis, we found that most behavioral tests are best at discriminating between injury and non-injury at ∼3–4 months post- rmTBI. This result was, however, skewed by our large quantity of MWM data compared with other measures. As can be seen in Figure 4, some behavioral tests had less clear global maxima. The review by Shultz and coworkers^4^ observed a variability in the injury detection capability of behavioral tests among the acute (≤ 3 days), subacute (≤ 3 months), and chronic (≥ 3 months) phases after rmTBI. Notably, for MWM, most studies found the greatest effects in the acute and subacute phases,^4^ whereas our study maximized the discrimination between injured and sham animals after 3–4 months (Figs. 4A and 5B). The maxima seen at this time point in our data might correspond to a window between heterogeneities after injury and full effect of neurodegeneration during later months.^42^ In the review by Shultz and coworkers,^4^ the OF test had a bimodal distribution, with most studies pointing toward better discrimination capability in the acute and chronic phases. This pattern was observed in our results (Figs. 4C and 5C), where the OF test for the circular arena had the highest discrimination capability during early and late time points. Our results regarding timing may be useful for the pre-clinical researcher planning new rmTBI-related experiments, but there is no straightforward conversion to the human timeline.^42^

### Importance of the MWM

In our study, the MWM was best able to discriminate between injured and uninjured animals. This result partially aligns with the previous prospective work by Zhao and coworkers,^8^ which also identifies the MWM as a key predictor. In their study, the reversal probe MWM (in which the platform was moved to the opposite quadrant without changing visual cues), outperformed the standard MWM. The reversal MWM was not tested in our study. Our results showed the best performance for the probe frequency as compared with the time spent in probe trials, but we achieved even better results when combining this with the hidden MWM trial during the final day of training. Zhao and coworkers^8^ performed all tests during the first 4 weeks after injury, whereas our results showed the best performances after ∼3–4 months. Although Zhao and coworkers^8^ investigated sham, mild, moderate, and severe TBI, they did not study repetitive mild injuries. All these variations in methodology could possibly account for the slight differences between their results and ours. The high performance of the MWM could be derived from the fact that it is sensitive to hippocampal deficits following neuronal dysfunction after injury, or because it contains aspects of the anxiety/depression and motor components in addition to its cognitive aspects.^28,43^ Because of concerns related to confounds, others have questioned the utility of the MWM;^44^ for example, changing the radius of the maze or the sex of the animals has been shown to change the outcome of the test substantially.^44^

### Other considerations when choosing the best test

Although our results indicate that the MWM is the best test to use in terms of capability to distinguish animals with rmTBI from uninjured animals, there are numerous other aspects that should be taken into account when choosing a test; for example, cost efficiency, specificity, and reliability.^45^ Although there are cheaper variations of the MWM, the standard procedure used in this analysis remains expensive and time consuming compared with other behavioral tests.^4,46^ Moreover, one should not blindly look at the overall performance of the MWM; each study has specific interventions addressing different parts of the pathophysiology, and tests measuring aspects other than spatial memory may be needed to answer the specific research questions. Tests that are less user- or laboratory-dependent, such as the rotarod, which automatically records the latency to fall from the rotating rod, has advantages of reliability as compared with the FST, in which an observer manually records immobility. Our results show that the FST performs very well, but recent attention has been given to the test when the animal-rights group People for the Ethical Treatment of Animals (PETA) urged United States institutions to stop supporting its use.^47^ In summary, a holistic and specific approach is needed when choosing appropriate behavioral tests.

### Behavioral tests performed best when combined

We found that behavioral tests could best distinguish injured from sham animals when these tests were combined. This aligns with previous results showing that individual tests do not consistently distinguish injured from sham animals.^4^ Composite scores of multiple behavioral tests have also outperformed single tests in previous animal studies.^8^ However, the compositions of these combined scores depend on the tests included in the respective studies, which are often arbitrarily chosen. Further, the performance measures used to assess the discriminative capability of different behavioral tests differ across different studies, creating difficulties in comparing the results. Our study uses the AUC as the performance measure, which is one of the most commonly used metrics of machine learning and is comparable across any predictive model.^9^

### Inclusion of multiple heterogeneous experiments or single homogeneous experiments

Our results suggest that a combination of multiple heterogeneous experiments might better predict behavioral outcomes than single homogeneous experiments. Many studies tend to discard non-homogeneous data, as these may create unnecessary noise, but we show that the noise created from multiple experiments (including different treatments) is less important than the larger sample size. Although the noise could indeed mask small significant differences and amount in type II errors, researchers performing retrospective pre-clinical TBI studies may still find it worthwhile to analyze larger heterogeneous data sets, as these can show effects of injury characteristics. Considering that many prospective animal studies are underpowered,^6^ retrospective analyses may serve as an ethically viable complement.

### Strengths and limitations

To the best of our knowledge, this is the first study to compare the capability of behavioral outcomes measured over 6 months to distinguish between rmTBI and sham injuries. With both supervised and unsupervised machine learning, we have shown that a combination of behavioral tests outperforms individual tests. We used logistical classification to achieve high interpretability in the supervised learning algorithm. In order to avoid overfitting, a dynamic approach in selecting potential predictors in the model was applied, where at least 10 mice were required per included predictor. The AUC was used as the performance measure, which makes our results comparable to those of future studies with similar data. Bootstrapping was also used, minimizing random errors in the algorithm. This study therefore used one of the simplest and most interpretable machine learning algorithms and makes use of state-of-the-art performance metrics and methodologies to identify the best behavioral test to detect rmTBI in mice.

There are important ethical advantages to combining previously collected data sets, which may reduce the number of animals needed for similar experiments, but there are also some pitfalls. Although <1200 mice were included in our study, the data were sparse, with large not-at-random missing chunks. The machine learning algorithms used required complete-case data, and the data were therefore often divided into complete-case subsets. This occasionally limited analyses to very few data, making it difficult to systematically adjust for treatment in the analyses without overfitting the models. Further, the subsetting introduced a bias as to which combinations of behavioral tests and experiments were possible to use simultaneously. Because data were collected retrospectively, only those behavioral tests and experiments that had been used in the original studies could be included. For example, some researchers might miss the comparison between the MWM and the swimming-independent Barnes maze.^48^ Further, combinations of tests not evaluated on the same mice could not be used in the same models. Also, our injury model utilizes rotational acceleration, which might limit generalizability to other models. Among the evaluated models, differences in number of mice and data structure possibly affected the results; larger sample sizes improve the model performance, and having a greater number of level 3 variables gives more options for picking optimal models. Although most tests included herein are common behavioral tests, there are numerous other behavioral tests that have not been evaluated in this analysis.^4^ Additionally, behavioral outcomes that have been more routinely tested in our laboratory may have higher experimenter consistency than those used occasionally. Tests with large amounts of data at multiple time points, such as the MWM, had by its data size better chances of performing well in machine learning algorithms than behavioral outcomes with less data, such as the novel object and location recognitions or the Y-maze. In summary, the limited sample size for each combination as well as the restricted number of combinations were the largest identified issues with our study design.

### Practical applications

Intervention studies that are based on behavioral outcomes require reliable tests that can identify a reversal of injury effects. Injured animals treated perfectly should have the same behavior as uninjured (sham) animals. Our results may therefore have implications for researchers in the field of pre-clinical rmTBI when planning experiments. Both choice of behavioral tests as well as timing can be guided by our findings. However, only limited conclusions can be drawn regarding pathophysiological implications of the optimal timing of behavioral tests. Although there is an expected correlation between the degree of pathology and the capability of a test to identify injury, the understanding of what behavioral tests actually measure is still limited.^4^ In this study, we used male mice that were predominantly 8 weeks old at first TBI/sham, which still have a developing brain. Our findings may therefore not be fully generalizable to females or mice of a different age (younger or older), as these have slightly different behavioral characteristics.^17,44,49^ Similarly, sex differences also exist in humans, where females have worse outcomes and show different structural alterations than males after mild TBI, although female hormones have a neuroprotective effect.^50^ Therefore, animal studies should ideally reflect sex differences and investigate both sexes. For our results to be used in this context, an analogous analysis to the one performed here should be conducted on female mice to confirm the applicability of our findings. Altogether, however, we believe that a majority of the signals observed in our data, despite large heterogeneity and use of male mice, are robust and could be used as a guide for future studies.

### Future studies

This is a retrospective study with data compiled from prior experiments, with a high degree of not-at-random missingness. Conclusions drawn from certain parts of the data, where complete cases include few mice, are weak and need to be strengthened by larger sample sizes. This could be done either retrospectively or prospectively. Retrospective large sample sizes could, for example, be achieved by creating databases in which researchers from multiple centers register raw data after publication. Prospective studies designed particularly for this purpose would give higher-quality data, because the same mice could undergo a plethora of tests. Prospective studies would, however, lead to additional and likely unnecessary use of animal subjects as compared with retrospective data gathering from previously completed studies. Larger data sets or data with a lower degree of missingness would open up the possibility of using more sophisticated machine learning tools, such as neural networks, and would also make it possible to establish a dose-response relationship of the repeated injuries and choice and timing of tests.

## Conclusion

Our study showed that a combination of behavioral tests outperforms individual tests when discriminating between animals with rmTBI and uninjured animals. We saw that the best timing for most individual behavioral tests is 3–4 months after first injury. The MWM was the behavioral test most capable of detecting injury effects. By identifying the optimal timing and choice of behavioral tests for rmTBI, we offer researchers guidance in how to optimize future experiments in the field.

## Supplementary Material

Supplemental data

Supplemental data

Supplemental data

## Supplementary Material

Supplementary Figures

Supplementary URL
