# Supplementary material for: Optimizing Choice and Timing of Behavioral Outcome Tests After Repetitive Mild Traumatic Brain Injury: A Machine Learning-Based Approach on Multiple Pre-Clinical Experiments
Source: J Neurotrauma. 2023 Aug 16;40(15-16):1762–78. doi: 10.1089/neu.2022.0486 (PMC10458377; doi:10.1089/neu.2022.0486)
Supplement: Supplemental data [file Suppl_URL.docx]

<https://lassaren.shinyapps.io/rmTBI_behavioral/>
