# Supplementary material for: Optimizing Choice and Timing of Behavioral Outcome Tests After Repetitive Mild Traumatic Brain Injury: A Machine Learning-Based Approach on Multiple Pre-Clinical Experiments
Source: J Neurotrauma. 2023 Aug 16;40(15-16):1762–78. doi: 10.1089/neu.2022.0486 (PMC10458377; doi:10.1089/neu.2022.0486)
Supplement: Supplemental data [file Suppl_Text.docx]

**Attachment 1**

**Treatments**

*External stimulus: flicker treatment*

The only external stimulus given was the flicker treatment (*n* = 60), theorized to restore the function of γ-aminobutyric acid (GABA)-ergic neurons,^1^ which release lower levels of GABA after rmTBI, and hence improve the otherwise poor cognitive function.^2^ No other mice (*n* = 1,097) received flicker treatment. The treatment consisted of a concurrent flash of light and sound at 40 interactions per second, which was theorized to restore the natural 40 Hz firing rate of these GABAergic neurons.^3^ The treatment lasted for 1 hour daily, while animals were in single housing. Light flashes were approximately 60 W bright, and sound was 25 to 30 dB loud. The mice were divided into three groups: the first group received 1 month of treatment starting 3 months after injury, the second group received 1 month of treatment starting immediately after injury, and the third group received 1 month of treatment starting 3 months after injury during their dark cycle, when mice are typically awake. These data have not been published previously.

*Genetic: tau knockouts*

There were 52 transgenic (28 hetero- and 24 homozygous) mice, lacking alleles of genes responsible for tau protein production; the other mice (*n* = 1,133) were not genetically modified. It has been shown that excessive tau aggregation following injury may be linked to behavioral deficits observed in mouse models.^4^ These data have not been published previously.

*Housing: single housing or enrichment*

There were two housing treatments — environmental enrichment (*n* = 149) and single housing (*n* = 12) — in addition to the normal cage (*n* = 996). Mice randomized to environmental enrichment were placed in groups of 10-15 animals in a Marlau™ cage,^5^ starting 3 days before injury and until they were sacrificed; the mice were only removed for behavioral testing. The cage has two floors and is designed to stimulate the animals by being equipped with running wheels, a climbing ladder, a slide tunnel and mazes. It has previously been shown that this type of environmental enrichment can promote cognitive activity in animal models.^6^ Mice randomized to single housing were placed alone in standard, translucent, plastic mouse cages. The remaining mice placed in normal cages were housed 5 mice per cage. A subset of the housing data has been published previously.^7^

*Intraperitoneal injection: cis P-tau, CRP, memantine, IgG or saline*

A small subgroup of mice received intraperitoneal treatment with one of the following active treatments — *cis* P-tau antibodies (*n* = 47), C-reactive protein (CRP; *n* = 6) and memantine (*n* = 65) — in addition to vehicle control (*n* = 118) in the forms of saline (*n* = 75) and IgG (*n* = 43). Mice randomized to *cis* P-tau antibody treatment received 200 µg intraperitoneally at various instances from 3 days before injury until behavioral tests were completed, as well as 20 µg intracerebroventricularly 15 min after injury; the details have been published previously.^8^ *Cis* P-tau antibodies inhibit apoptosis by preventing *cis* P-tau from disrupting mitochondrial transport and axonal microtubule networks.^8^ Injected monomeric CRP has been shown to promote dementia after ischemia in mice, which is theorized to be applicable to experimental TBI.^9^ Mice randomized to memantine treatment received a dose of 10 mg/kg (Tocris, Minneapolis, MN, USA) in 0.2 ml of saline 30 min after the last injury. Memantine is a medication against Alzheimer’s disease approved by both American and European regulatory bodies,^10^ and in this context it was used to target N-methyl-D-aspartate (NMDA) receptor-mediated glutamatergic toxicity, thereby mitigating rmTBI-induced neurologic deficits.^11^ IgG was used as a vehicle control for the *cis* P-tau antibodies, while saline was used as a vehicle control for the other smaller molecules. Parts of the data for the intraperitoneally injected treatments have been previously published.^8,11-13^

*Intranasal delivery: anti-CD3 or IgG*

The only active treatment given via this route was anti-CD3 (cluster of differentiation 3, *n* = 23), which is an antibody that binds to an antigen that is partially responsible for T cell activation; the remaining mice with intranasal treatments (*n* = 24) received the IgG vehicle. It has been theorized that preventing some of this activation would mitigate a hyperactive immune response to TBI. The treated animals received doses of 0.5 µg of anti-CD3 antibody intranasally in a 10 µl solution, starting at 6 hours post injury, and extending throughout their behavioral tests. For the first 9 days, doses were given daily and henceforth thrice weekly until sacrifice. These data have not been published previously.

*Oral (via water): memantine or saline*

Memantine (*n* = 49) and saline (*n* = 48) were given orally via water at doses of 20 mg/kg to mice. The hypothesized effect was similar to that given intraperitoneally. These data have not been published previously.

**Behavioral outcomes**

*Cognitive deficits*

Morris water maze, novel object recognition, novel location recognition and Y-maze were used to assess aspects of cognitive deficits.

Morris water maze

The Morris water maze has been developed to study spatial learning and memory in rodents.^14^ Methods and a subset of data have been previously published.^7,8,12,13,15-18^ The maze consisted of a white cylindrical tank (83 cm diameter, 60 cm deep) with water filled to 29 cm depth, at ~24-25 °C. Intra- and extra-maze cues were highly visual, forming four distinct quadrants. A round, clear, Plexiglas platform (10 cm in diameter) was placed 0.5-1 cm below the surface of the water in the southwest quadrant 15 cm from the wall. There were four types of trials: hidden, visual, probe and probe frequency trials. In hidden and visual platform trials, each mouse was randomized four times without replacement to one of four starting locations (north, south, east, or west), such that each trial consisted of two sub-trials starting from opposite, alternating quadrants. Initially, mice were placed in the container facing the wall, after which they were given 90 sec to find the location of the hidden platform, mount the platform, and remain on it for 5-10 sec. All mice were rewarded by drying under a heat lamp until their next run. The time until the mouse mounted the platform (i.e. escape latency) was measured and recorded. Mice that failed to mount the platform within the allotted time (80-90 sec) were gently guided by the experimenter to the platform and allowed to remain there for 10 sec; for these mice, escape latency was recorded as 80-90 sec, depending on the allotted time for the trial. Each mouse conducted a maximum of two trials per day, each consisting of four runs, with 20-45 min break between trials, allowing time for acquisition. After completion of up to five hidden platform trials, up to two visual platform trials were conducted. During these, a red reflector was used to mark the top of the target platform. Similarly, probe trials were conducted after completion of hidden trials. Here, mice were placed in the tank with the platform removed and were given 60 sec to explore the tank. EthoVision® 9-11.5 (Noldus Information Technology BV, Wageningen, Netherlands) software tracked time spent in the target quadrant where the platform was previously located. The number of times mice passed the previous location of the platform was recorded as probe frequency. In some cases, repeated Morris water maze testing was conducted several weeks later, during which the platform was moved to a different quadrant than that used previously. Rapidly locating the platform or spending much time in its previous location is associated with good spatial learning and memory.^14^

Novel object and location recognition tests

The novel object and location recognition tests have been developed to study working memory in rodents.^19^ Methods and a subset of data have been previously published.^12^ Both tests consisted of an open-field box (44 × 44 cm). During 3 days, mice were habituated for 5 min daily via free exploration in the arena. On test day, the animals were allowed to become familiarized with three identical objects (consisting of same-color Lego® sets) placed in a fixed location inside the field for 6 min. The time spent inspecting the individual objects was recorded using EthoVision® XT (Noldus Information Technology BV, Wageningen, Netherlands). Directly after the familiarization session, mice were removed and one of the objects was either replaced by a novel object or moved to a novel location inside the arena. The mice were then allowed an additional 3 min for exploration, and time spent at the novel object or location was recorded as a fraction of total time spent in the arena. The floor was covered with sawdust (1 cm deep, used and saturated with the odor of the animals) during habituation, familiarization and test sessions. A large fraction of time spent at the novel object or location is associated with good working memory.^19^

Y-maze

Spontaneous alternation in a Y-maze has been used to study spatial working memory and other cognitive aspects in rodents.^20-22^ Methods and data have not previously been published, but the protocol used is in principle similar to the one described by Albayram et al.^23^. The maze consisted of a Y-shaped container with three identical arms oriented at 120° angles from each other. A mouse was placed in the center for 5 min and the movement was recorded using EthoVision® XT (Noldus Information Technology BV, Wageningen, Netherlands). Finally, the fraction of alternation behavior was calculated using the total number of alternations, defined as consecutive entries into all three arms, and the total number of arm entries. A large alternation fraction is associated with good working memory.^22^

*Anxiety and depression*

Elevated plus maze, open field test, forced swim test, light-dark box, tail suspension test and sucrose preference test were used to assess aspects of anxiety and depression.

Elevated plus maze

The elevated plus maze has been developed to study anxiety-like behavior in rodents.^24^ Methods and a subset of data have been previously published.^7,8,13,17,18^ The maze consisted of a platform with two open and two closed (walled) arms (Lafayette Instrument, Lafayette, IN, USA). Each arm measured 30 × 5 cm and radiated from a central decision zone, forming a plus shape. The entire maze was raised 85 cm above the floor. Mice were placed in the decision area, facing a closed arm, and were allowed to explore the apparatus for 5 min. Total time spent in the open arm, closed arm and decision zone was recorded using EthoVision® 11.5 (Noldus Information Technology BV, Wageningen, Netherlands). Only The apparatus was cleaned between tests with a weak ethanol solution or Clidox® (1:18:1 Clidox® base / water / Clidox® activator) and dried. The more time spent in open arms is associated with lower levels of anxiety.^24^

Open field test

The open field test has been developed to study anxiety-like behavior in rodents,^25^ but is often also used to measure locomotor activity.^26^ These methods and a subset of data have been previously published.^7,13,18^ The open field consisted of either an opaque, plastic cylinder (45 cm diameter and 20 cm height) or a transparent cuboid (40 × 40 cm base and 30 cm height). It was placed inside a transparent plastic box and in an enclosed chamber with dimmed lights to prevent distraction. At the beginning of each trial, mice were placed in a certain part of the edge of the arena facing the wall. The circular arena was virtually divided into three distinct concentric circular sections: an inner circle with a 10 cm radius (area of 314 cm^2^) and two annuli with boundaries at radii of 10, 20 and 30 cm (areas of 942 cm^2^ and 1,571 cm^2^, respectively). The square arena was virtually divided into equal square-shaped peripheral and central areas (each 800 cm^2^). Mice were given 10 min to explore the arena. Fraction of time spent in each of the regions was recorded using EthoVision® (Noldus Information Technology BV, Wageningen, Netherlands) for circular fields and MotorMonitor® II (Kinder Scientific, Poway, CA, USA) for square fields. For square fields, distance travelled was also recorded. The arena was cleaned in between each trial (Peroxigard™ wipes, Oakville, ON, Canada). More time spent in the central areas of the open field is associated with low levels of anxiety.^26^

Forced swim test

The forced swim test has been developed to study depression-like behavior in rodents.^27^ Methods and a subset of data have been previously published.^7^ A glass cylinder (18 cm diameter and 40 cm height) was filled with water up to 30 cm at 25°C. Mice were placed individually in the container for 5 min, and the session was recorded with a video camera. Once a mouse was floating and making only the necessary movements to keep its head above the water surface, it was considered immobile. An experimenter, blinded to the animal treatments, observed the videotapes and recorded immobility time as a fraction of total time in the water. The experiment was repeated for up to 3 days. Larger fraction of immobility is associated with depressant-like behavior.^27^

Light-dark box

The light-dark box has been developed to study anxiety-like behavior in rodents,^28^ but is often also considered to measure exploratory behavior.^29^ Methods and data have not previously been published, but the protocol used is similar to the one described by Takao & Miyakawa.^30^ An open box was brightly lit (30-50 lux) and connected to a dark chamber, separated by a wall. Mice were placed in the center of the chamber. Total time in each box and distance was recorded for 5 minutes using EthoVision® XT (Noldus Information Technology BV, Wageningen, Netherlands). Fewer transitions between the arms and less locomotor activity is associated with anxiety-like behavior.^28^

Tail suspension test

The tail suspension test has been developed to study depressant-like behavior in rodents.^31^ Methods and data have not previously been published, but the protocol used is in principle similar to the one described originally by Steru et al.^31^ Mice were suspended by the tail for 6 min. The fraction of time spent immobile was recorded using EthoVision® XT (Noldus Information Technology BV, Wageningen, Netherlands). Larger fraction of immobility is associated with depressant-like behavior.^31^

Sucrose preference test

The sucrose preference test has been developed to study depressant-like behavior in rodents by measuring hedonic deficit.^32^ Methods and data have not previously been published, but the protocol used is similar to the one described by Jenniches et al.^33^ Mice were placed in a cage with two sipper tubes, one of which contained normal drinking water and the other with 2 % sucrose added. Bottles were switched daily to avoid side bias. The fraction of daily sucrose solution consumption and total daily intake of fluid was recorded for days 2-4 of experiment. Lower intake of sucrose solution is associated with depressant-like behavior.^32^

*Motor deficits*

Rotarod test was used to assess the motor deficits. The previously described open field tests are also often considered to have a motor component.^34^

Rotarod

The accelerating rotarod test has been developed to study neurological deficit in rodents.^35,36^ Methods and a subset of data have been previously published.^7,12,13,18^ The rotarod consisted of a rotating cylinder, 4 cm in diameter, which was suspended within a box. Mice were trained and habituated during the first day by being placed on the rotating drum (4 rpm) for 5 min and immediately put back if they fell off. Testing was performed four times per day for the following 2 days. Mice were placed on the rotating drum (4 rpm) for 10 sec to acclimate, after which the rod was accelerated by 0.1 rpm/sec to a maximum of 40 rpm. Latency to fall was recorded during a maximum of 10 min testing, and averaged daily. Animals rested at least 5 min between trials to avoid exhaustion. Shorter latency to fall is associated with greater neurological deficit.^35,36^

**References**

1. Xu Y, Zhao M, Han Y, et al. GABAergic Inhibitory Interneuron Deficits in Alzheimer's Disease: Implications for Treatment. Front Neurosci 2020;14(660, doi:10.3389/fnins.2020.00660

2. Kim GH, Kang I, Jeong H, et al. Low Prefrontal GABA Levels Are Associated With Poor Cognitive Functions in Professional Boxers. Front Hum Neurosci 2019;13(193, doi:10.3389/fnhum.2019.00193

3. Pinault D, Deschênes M. Control of 40-Hz firing of reticular thalamic cells by neurotransmitters. Neuroscience 1992;51(2):259-68, doi:10.1016/0306-4522(92)90313-q

4. Flunkert S, Hierzer M, Löffler T, et al. Elevated levels of soluble total and hyperphosphorylated tau result in early behavioral deficits and distinct changes in brain pathology in a new tau transgenic mouse model. Neurodegener Dis 2013;11(4):194-205, doi:10.1159/000338152

5. Fares RP, Belmeguenai A, Sanchez PE, et al. Standardized environmental enrichment supports enhanced brain plasticity in healthy rats and prevents cognitive impairment in epileptic rats. PLoS One 2013;8(1):e53888, doi:10.1371/journal.pone.0053888

6. Bondi CO, Klitsch KC, Leary JB, et al. Environmental enrichment as a viable neurorehabilitation strategy for experimental traumatic brain injury. J Neurotrauma 2014;31(10):873-88, doi:10.1089/neu.2014.3328

7. Liu X, Qiu J, Alcon S, et al. Environmental Enrichment Mitigates Deficits after Repetitive Mild Traumatic Brain Injury. J Neurotrauma 2017;34(16):2445-2455, doi:10.1089/neu.2016.4823

8. Kondo A, Shahpasand K, Mannix R, et al. Antibody against early driver of neurodegeneration cis P-tau blocks brain injury and tauopathy. Nature 2015;523(7561):431-436, doi:10.1038/nature14658

9. Slevin M, Matou S, Zeinolabediny Y, et al. Monomeric C-reactive protein--a key molecule driving development of Alzheimer's disease associated with brain ischaemia? Sci Rep 2015;5(13281, doi:10.1038/srep13281

10. Morant AV, Vestergaard HT, Lassen AB, et al. US, EU, and Japanese Regulatory Guidelines for Development of Drugs for Treatment of Alzheimer's Disease: Implications for Global Drug Development. Clin Transl Sci 2020;13(4):652-664, doi:10.1111/cts.12755

11. Ma G, Liu C, Hashim J, et al. Memantine Mitigates Oligodendrocyte Damage after Repetitive Mild Traumatic Brain Injury. Neuroscience 2019;421(152-161, doi:10.1016/j.neuroscience.2019.10.016

12. Albayram O, Kondo A, Mannix R, et al. Cis P-tau is induced in clinical and preclinical brain injury and contributes to post-injury sequelae. Nat Commun 2017;8(1):1000, doi:10.1038/s41467-017-01068-4

13. Mei Z, Qiu J, Alcon S, et al. Memantine improves outcomes after repetitive traumatic brain injury. Behav Brain Res 2018;340(195-204, doi:10.1016/j.bbr.2017.04.017

14. Morris R. Developments of a water-maze procedure for studying spatial learning in the rat. J Neurosci Methods 1984;11(1):47-60, doi:10.1016/0165-0270(84)90007-4

15. Meehan WP, Zhang J, Mannix R, et al. Increasing recovery time between injuries improves cognitive outcome after repetitive mild concussive brain injuries in mice. Neurosurgery 2012;71(4):885-91, doi:10.1227/NEU.0b013e318265a439

16. Mannix R, Meehan WP, Mandeville J, et al. Clinical correlates in an experimental model of repetitive mild brain injury. Ann Neurol 2013;74(1):65-75, doi:10.1002/ana.23858

17. Mannix R, Berkner J, Mei Z, et al. Adolescent Mice Demonstrate a Distinct Pattern of Injury after Repetitive Mild Traumatic Brain Injury. J Neurotrauma 2017;34(2):495-504, doi:10.1089/neu.2016.4457

18. Mannix R, Berglass J, Berkner J, et al. Chronic gliosis and behavioral deficits in mice following repetitive mild traumatic brain injury. J Neurosurg 2014;121(6):1342-50, doi:10.3171/2014.7.JNS14272

19. Ennaceur A, Delacour J. A new one-trial test for neurobiological studies of memory in rats. 1: Behavioral data. Behav Brain Res 1988;31(1):47-59, doi:10.1016/0166-4328(88)90157-x

20. Tolman EC. Purpose and cognition: the determiners of animal learning. Psychological review 1925;32(4):285-297, doi:10.1037/h0072784

21. Hughes RN. The value of spontaneous alternation behavior (SAB) as a test of retention in pharmacological investigations of memory. Neurosci Biobehav Rev 2004;28(5):497-505, doi:10.1016/j.neubiorev.2004.06.006

22. Kraeuter A-K, Guest PC, Sarnyai Z. The Y-Maze for Assessment of Spatial Working and Reference Memory in Mice. In: Pre-Clinical Models: Techniques and Protocols. (Guest PC. ed.) Springer New York: New York, NY; 2019; pp. 105-111.

23. Albayram O, MacIver B, Mathai J, et al. Traumatic Brain Injury-related voiding dysfunction in mice is caused by damage to rostral pathways, altering inputs to the reflex pathways. Sci Rep 2019;9(1):8646, doi:10.1038/s41598-019-45234-8

24. Handley SL, Mithani S. Effects of alpha-adrenoceptor agonists and antagonists in a maze-exploration model of 'fear'-motivated behaviour. Naunyn Schmiedebergs Arch Pharmacol 1984;327(1):1-5, doi:10.1007/BF00504983

25. Hall CS. Emotional behavior in the rat. I. Defecation and urination as measures of individual differences in emotionality. Journal of Comparative Psychology 1934;18(3):385-403, doi:10.1037/h0071444

26. Kraeuter A-K, Guest PC, Sarnyai Z. The Open Field Test for Measuring Locomotor Activity and Anxiety-Like Behavior. In: Pre-Clinical Models: Techniques and Protocols. (Guest PC. ed.) Springer New York: New York, NY; 2019; pp. 99-103.

27. Porsolt RD, Le Pichon M, Jalfre M. Depression: a new animal model sensitive to antidepressant treatments. Nature 1977;266(5604):730-2, doi:10.1038/266730a0

28. Crawley J, Goodwin FK. Preliminary report of a simple animal behavior model for the anxiolytic effects of benzodiazepines. Pharmacol Biochem Behav 1980;13(2):167-70, doi:10.1016/0091-3057(80)90067-2

29. Bourin M, Hascoët M. The mouse light/dark box test. Eur J Pharmacol 2003;463(1-3):55-65, doi:10.1016/s0014-2999(03)01274-3

30. Takao K, Miyakawa T. Light/dark transition test for mice. J Vis Exp 2006;1):104, doi:10.3791/104

31. Steru L, Chermat R, Thierry B, et al. The tail suspension test: a new method for screening antidepressants in mice. Psychopharmacology (Berl) 1985;85(3):367-70, doi:10.1007/BF00428203

32. Katz RJ. Animal model of depression: pharmacological sensitivity of a hedonic deficit. Pharmacol Biochem Behav 1982;16(6):965-8, doi:10.1016/0091-3057(82)90053-3

33. Jenniches I, Ternes S, Albayram O, et al. Anxiety, Stress, and Fear Response in Mice With Reduced Endocannabinoid Levels. Biol Psychiatry 2016;79(10):858-868, doi:10.1016/j.biopsych.2015.03.033

34. Shultz SR, McDonald SJ, Corrigan F, et al. Clinical Relevance of Behavior Testing in Animal Models of Traumatic Brain Injury. J Neurotrauma 2020;37(22):2381-2400, doi:10.1089/neu.2018.6149

35. Dunham NW, Miya TS. A note on a simple apparatus for detecting neurological deficit in rats and mice. J Am Pharm Assoc Am Pharm Assoc 1957;46(3):208-9, doi:10.1002/jps.3030460322

36. Jones BJ, Roberts DJ. A rotarod suitable for quantitative measurements of motor incoordination in naive mice. Naunyn Schmiedebergs Arch Exp Pathol Pharmakol 1968;259(2):211, doi:10.1007/BF00537801
