# Supplementary material for: Optimizing Choice and Timing of Behavioral Outcome Tests After Repetitive Mild Traumatic Brain Injury: A Machine Learning-Based Approach on Multiple Pre-Clinical Experiments
Source: J Neurotrauma. 2023 Aug 16;40(15-16):1762–78. doi: 10.1089/neu.2022.0486 (PMC10458377; doi:10.1089/neu.2022.0486)
Supplement: Supplemental data [file Suppl_Figures.pdf]

Attachment 2:

One of the combinations of each *k*-means clusters for 2-15 as of Figure 6A, as well as the ground truth (TBI or sham). The order of the figures is ascending from 2 variables to 15 variables. The variables displayed were randomly selected, and serve as illustrative examples.

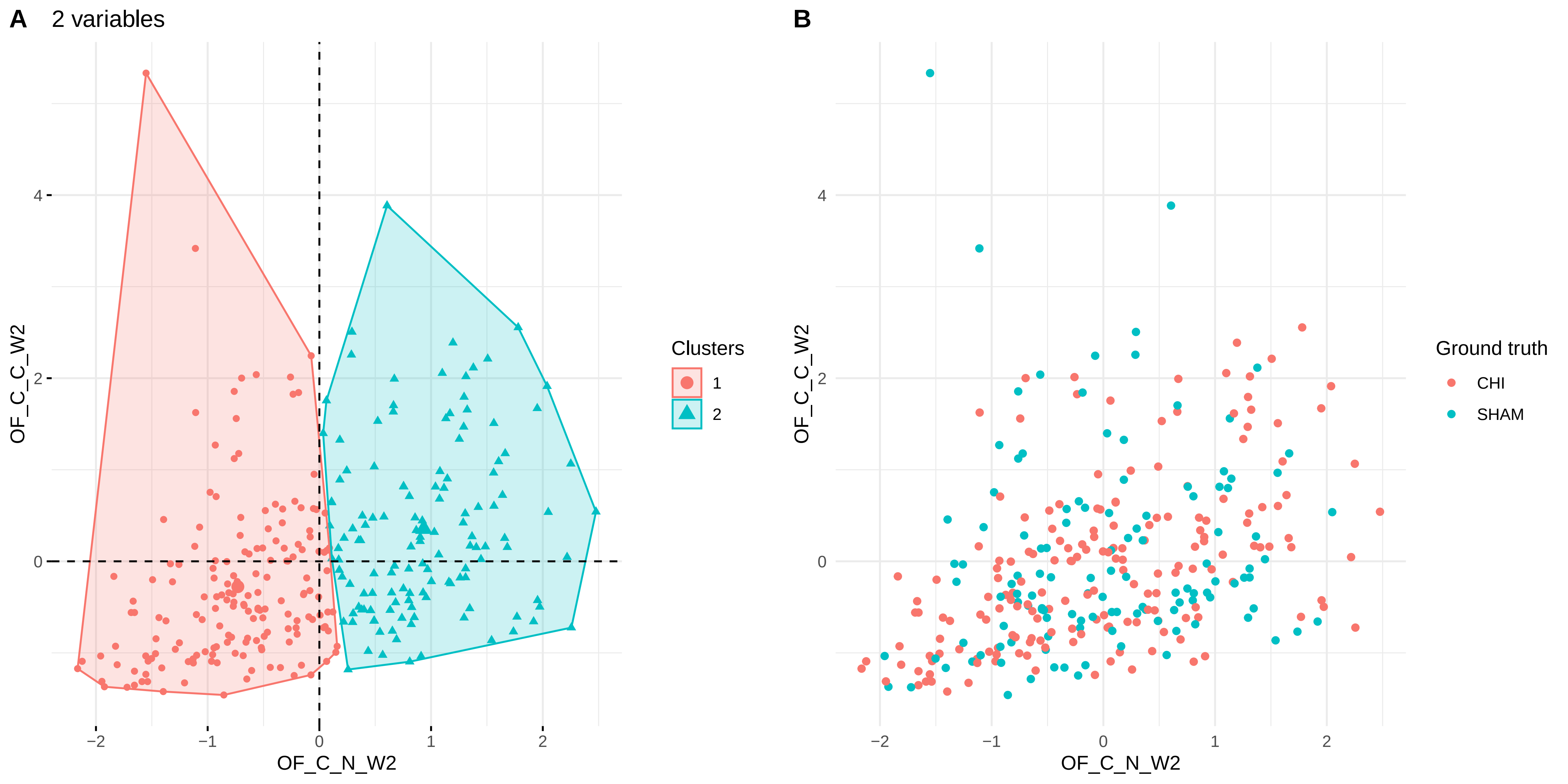
A
2 variables
OF_C_C_W2
OF_C_N_W2
Clusters
1
2
B
OF_C_C_W2
OF_C_N_W2
Ground truth
CHI
SHAM

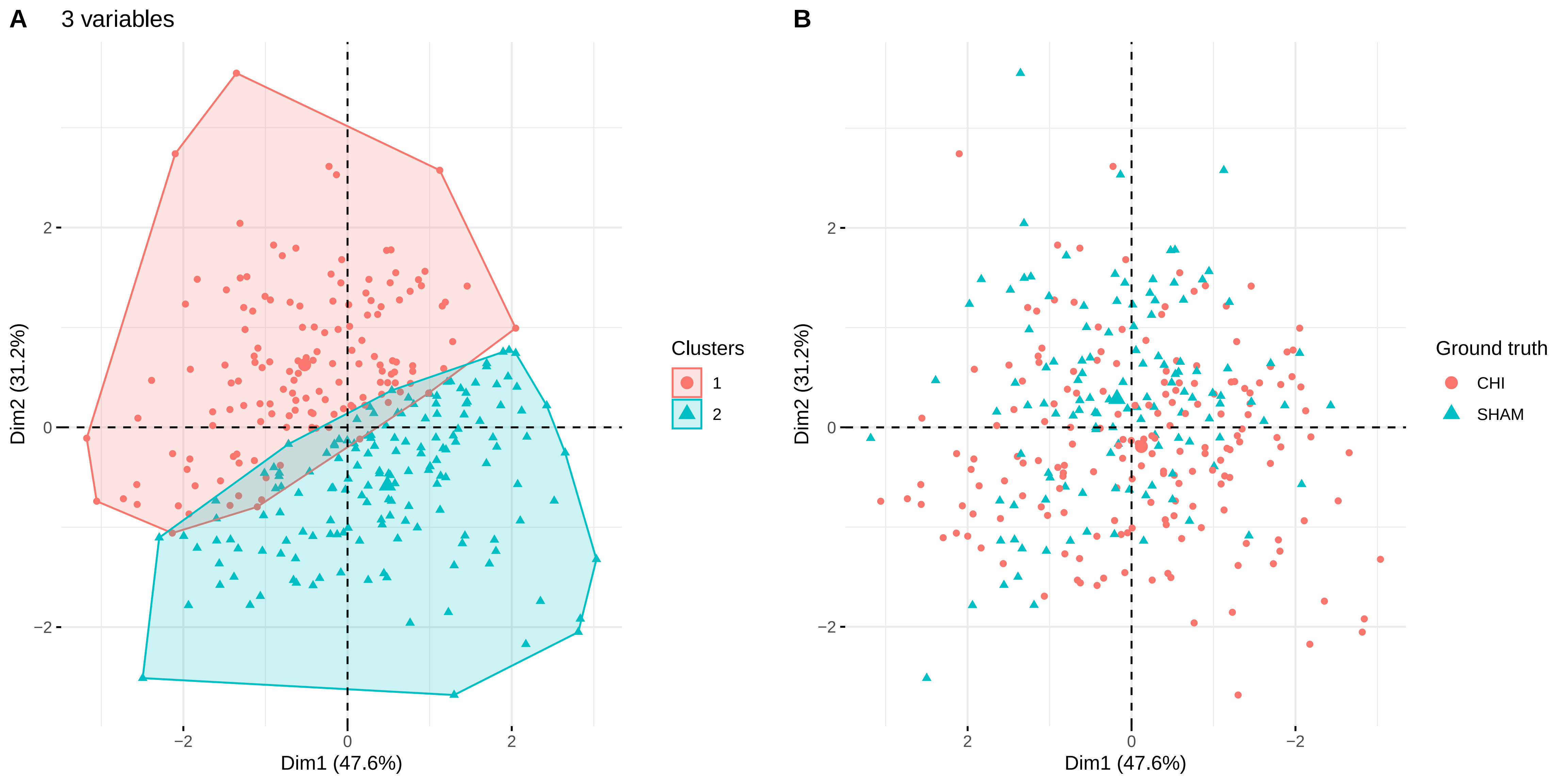

A
3 variables
Dim2 (31.2%)
Dim1 (47.6%)
Clusters
1
2
B
Dim2 (31.2%)
Dim1 (47.6%)
Ground truth
CHI
SHAM

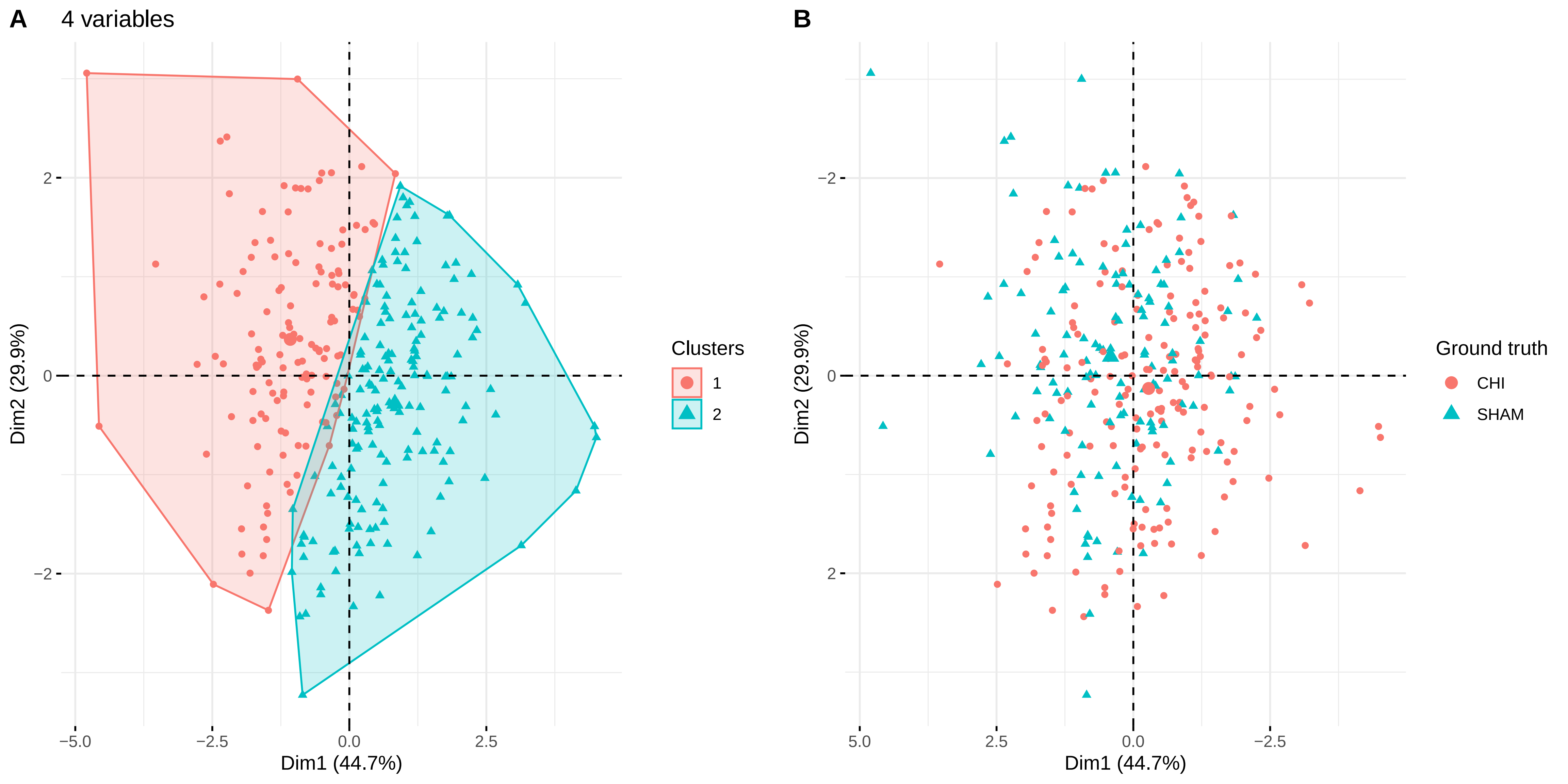

A
4 variables
Clusters
1
2
Dim2 (29.9%)
Dim1 (44.7%)
B
Ground truth
CHI
SHAM
Dim2 (29.9%)
Dim1 (44.7%)

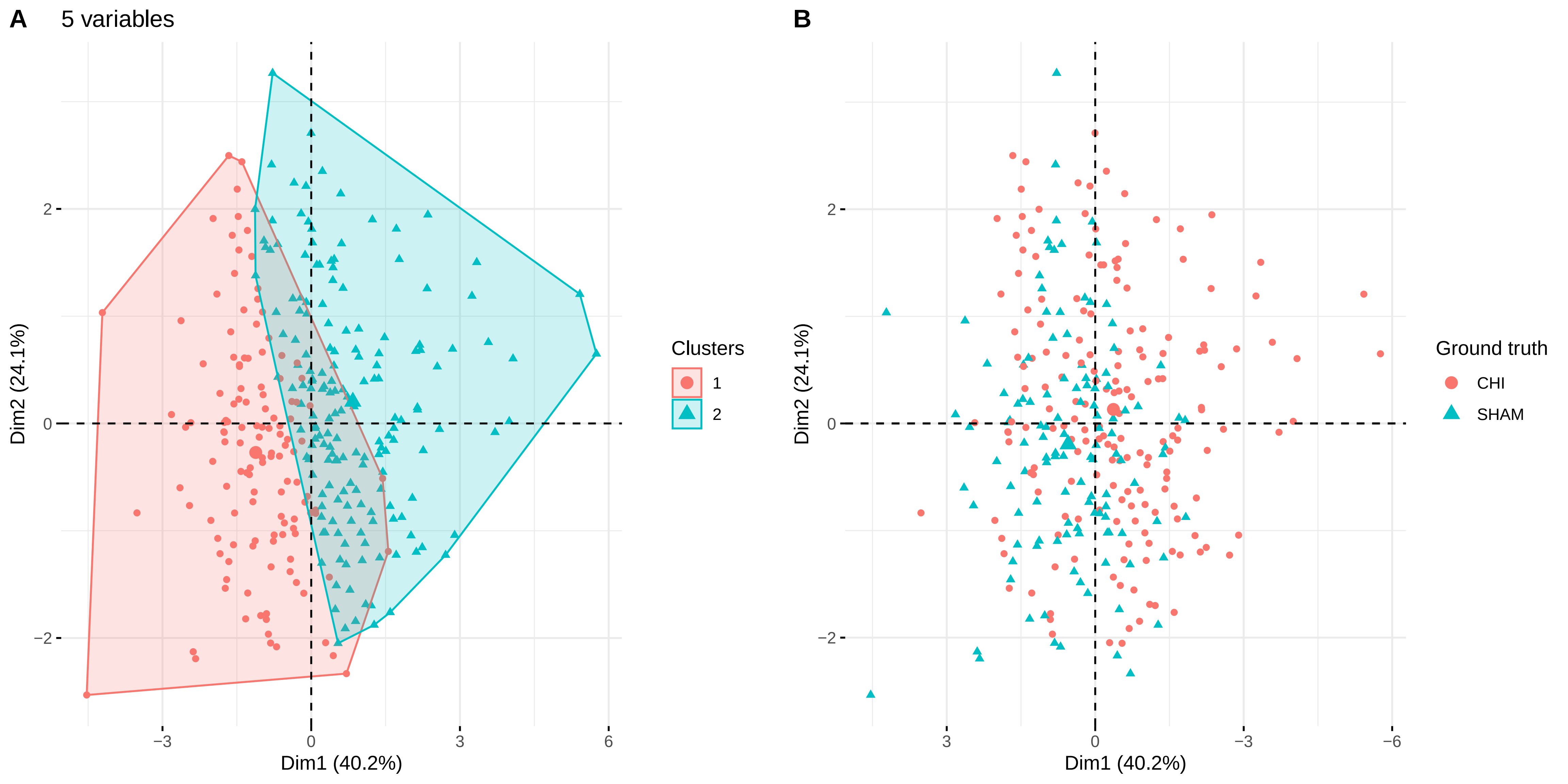

A
5 variables
Dim2 (24.1%)
Dim1 (40.2%)
Clusters
1
2
B
Dim2 (24.1%)
Dim1 (40.2%)
Ground truth
CHI
SHAM

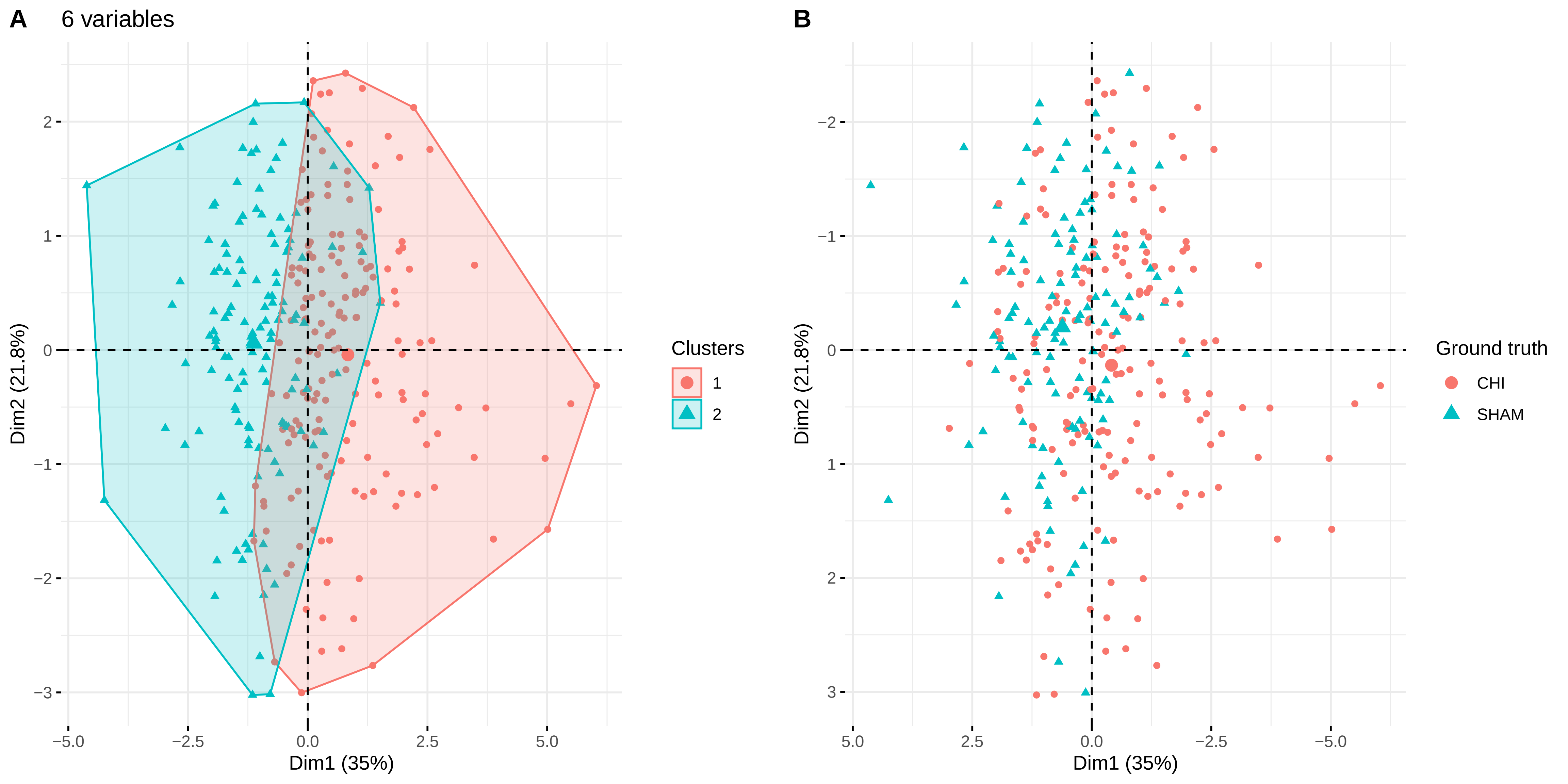
A
6 variables
B
Clusters
1
2
Ground truth
CHI
SHAM
Dim1 (35%)
Dim2 (21.8%)
Dim1 (35%)
Dim2 (21.8%)

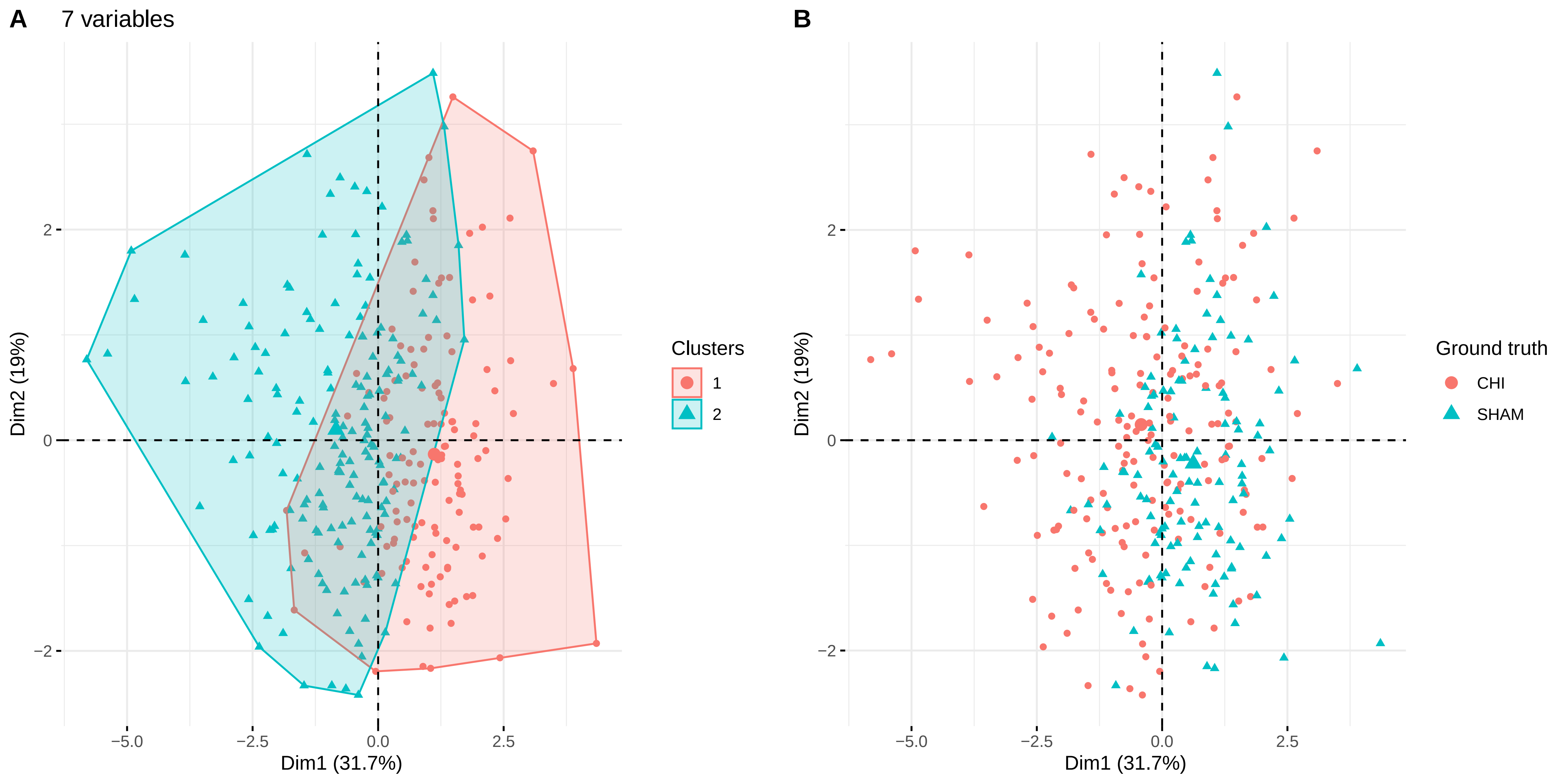

A
7 variables
B
Dim2 (19%)
Dim1 (31.7%)
−5.0
−2.5
0.0
2.5
2
0
−2
Clusters
1
2
Ground truth
CHI
SHAM

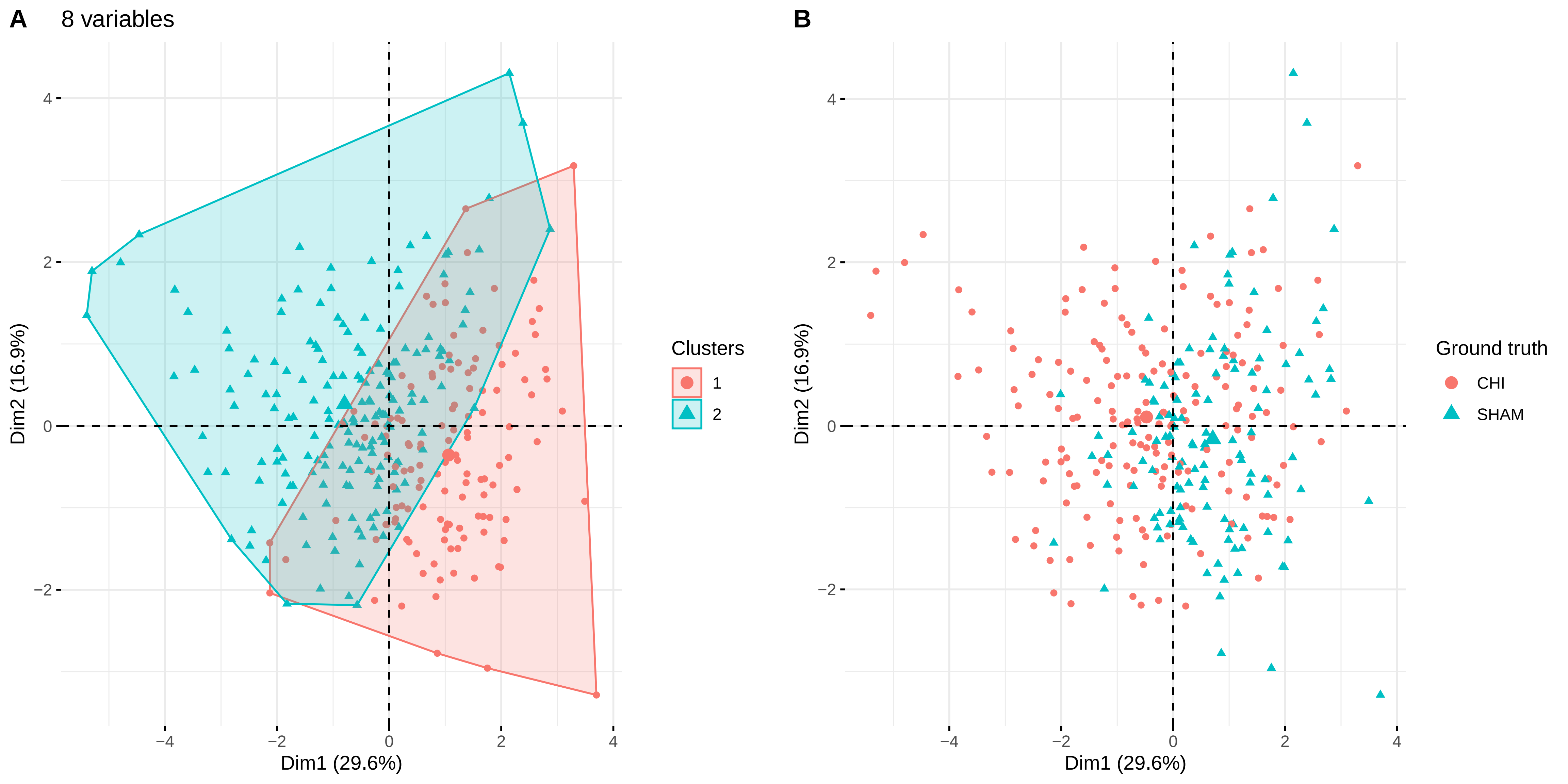
A
8 variables
Dim2 (16.9%)
Dim1 (29.6%)
Clusters
1
2
B
Ground truth
CHI
SHAM
−4
−2
0
2
4

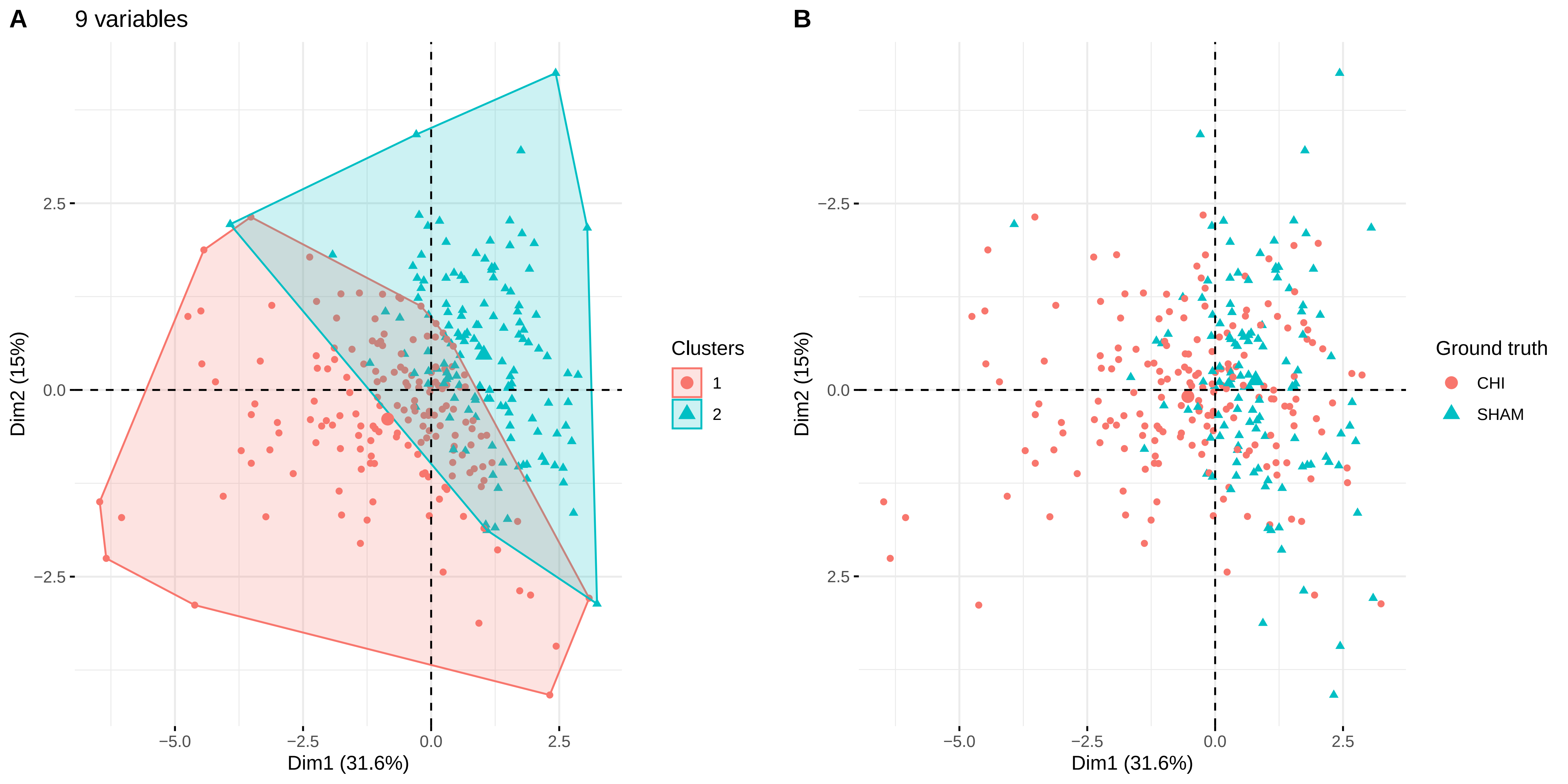

A
9 variables
Dim1 (31.6%)
Dim2 (15%)
−5.0
−2.5
0.0
2.5
Clusters
1
2
B
Ground truth
CHI
SHAM

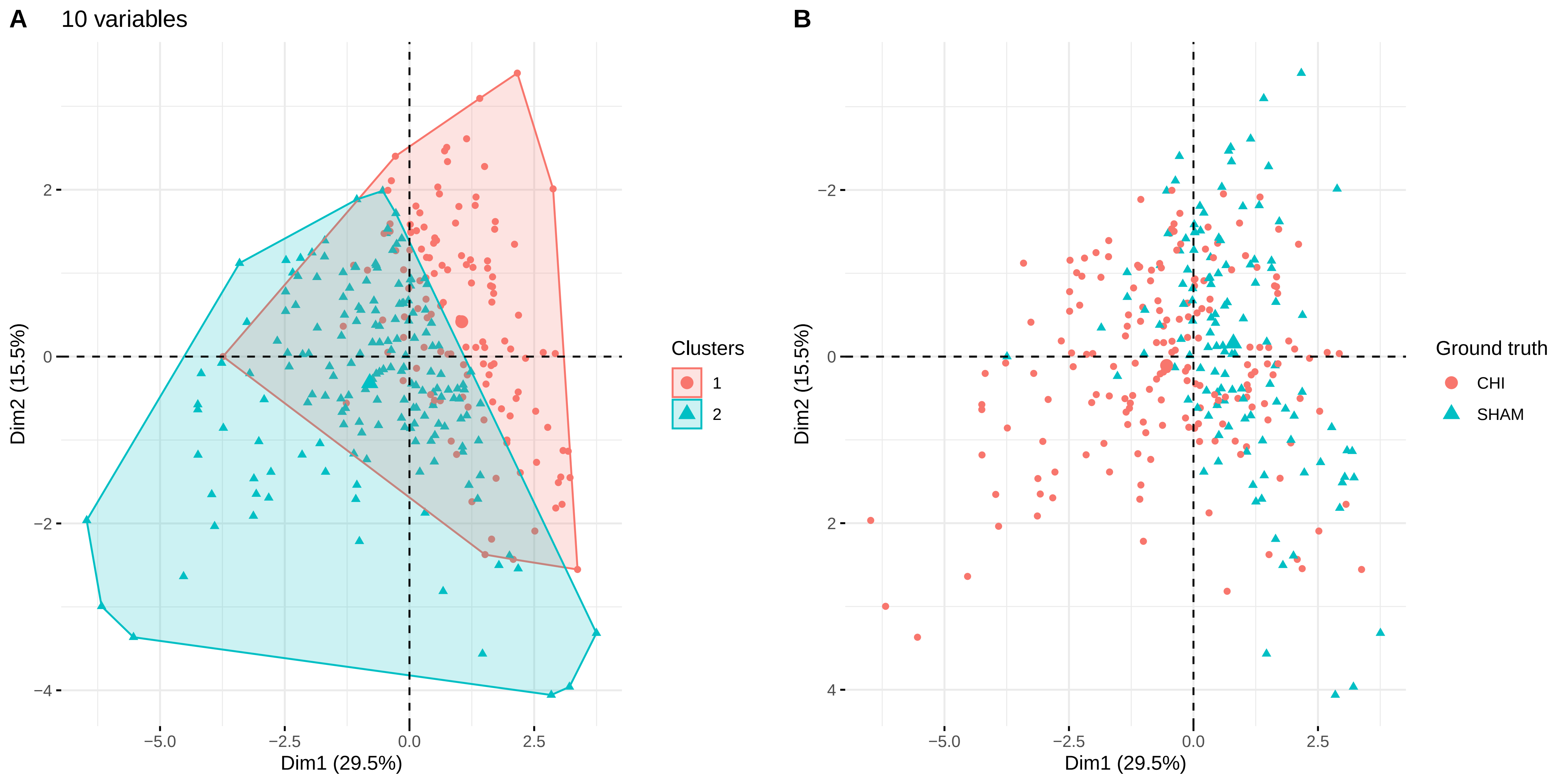
A
10 variables
Dim2 (15.5%)
Dim1 (29.5%)
Clusters
1
2
B
Ground truth
CHI
SHAM
−5.0
−2.5
0.0
2.5
2
0
−2
−4
4

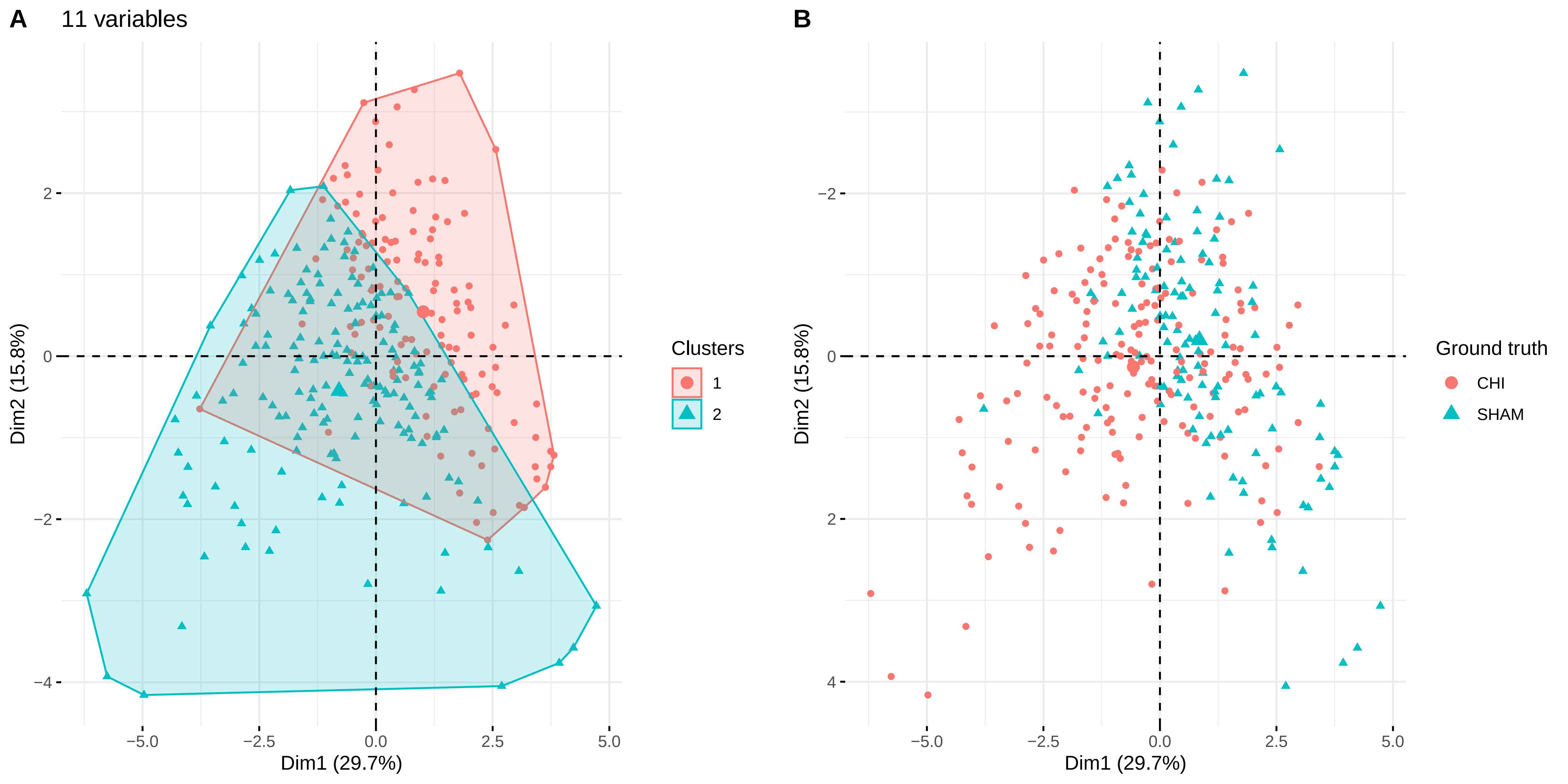
A
11 variables
Dim2 (15.8%)
Dim1 (29.7%)
−5.0
−2.5
0.0
2.5
5.0
2
0
−2
−4
Clusters
1
2
B
Dim2 (15.8%)
Dim1 (29.7%)
−2
0
2
4
Ground truth
CHI
SHAM

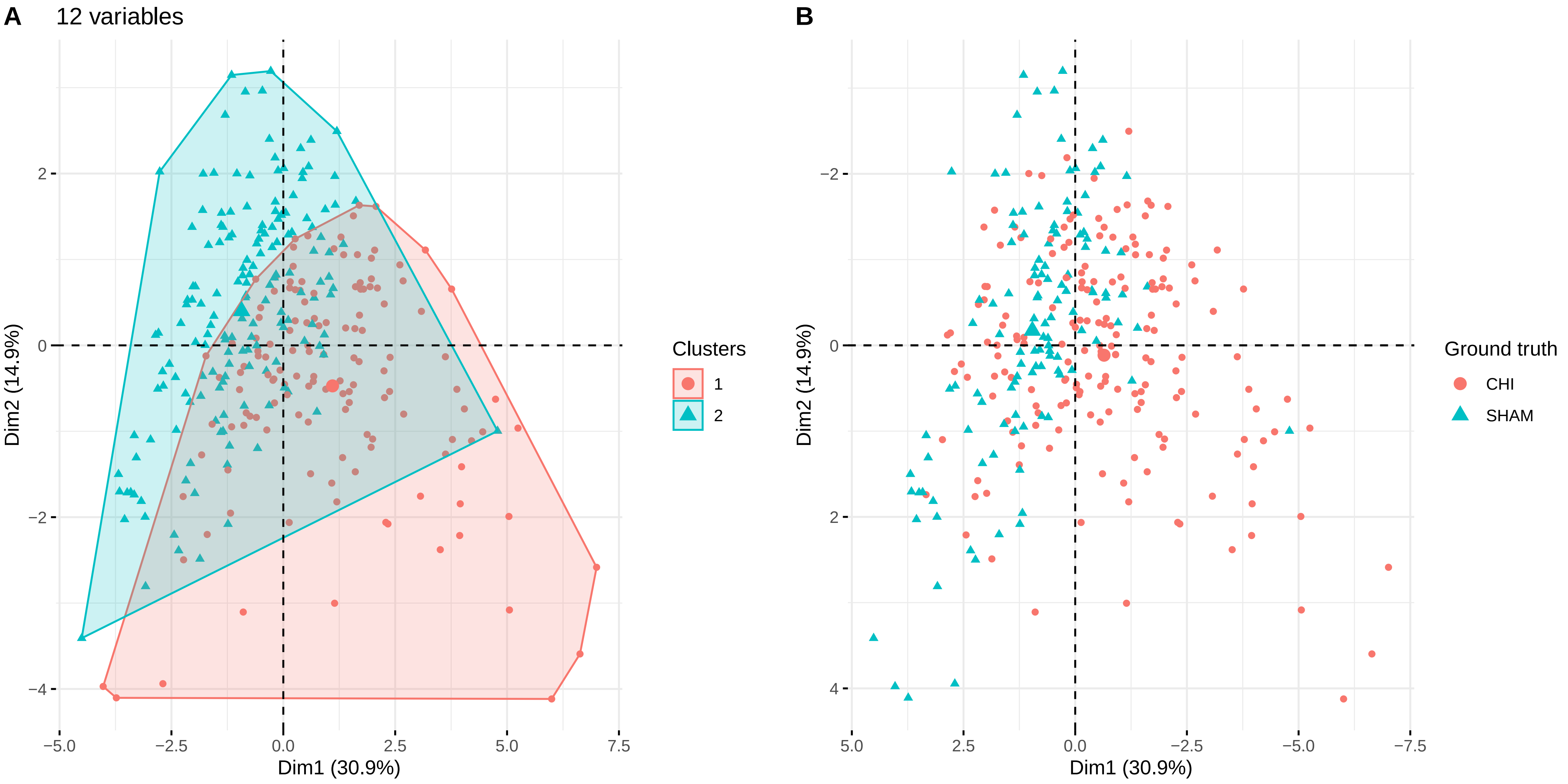
A
12 variables
Clusters
1
2
Dim1 (30.9%)
Dim2 (14.9%)
−5.0
−2.5
0.0
2.5
5.0
7.5
2
0
−2
−4
B
Ground truth
CHI
SHAM
Dim1 (30.9%)
Dim2 (14.9%)
5.0
2.5
0.0
−2.5
−5.0
−7.5
−2
0
2
4

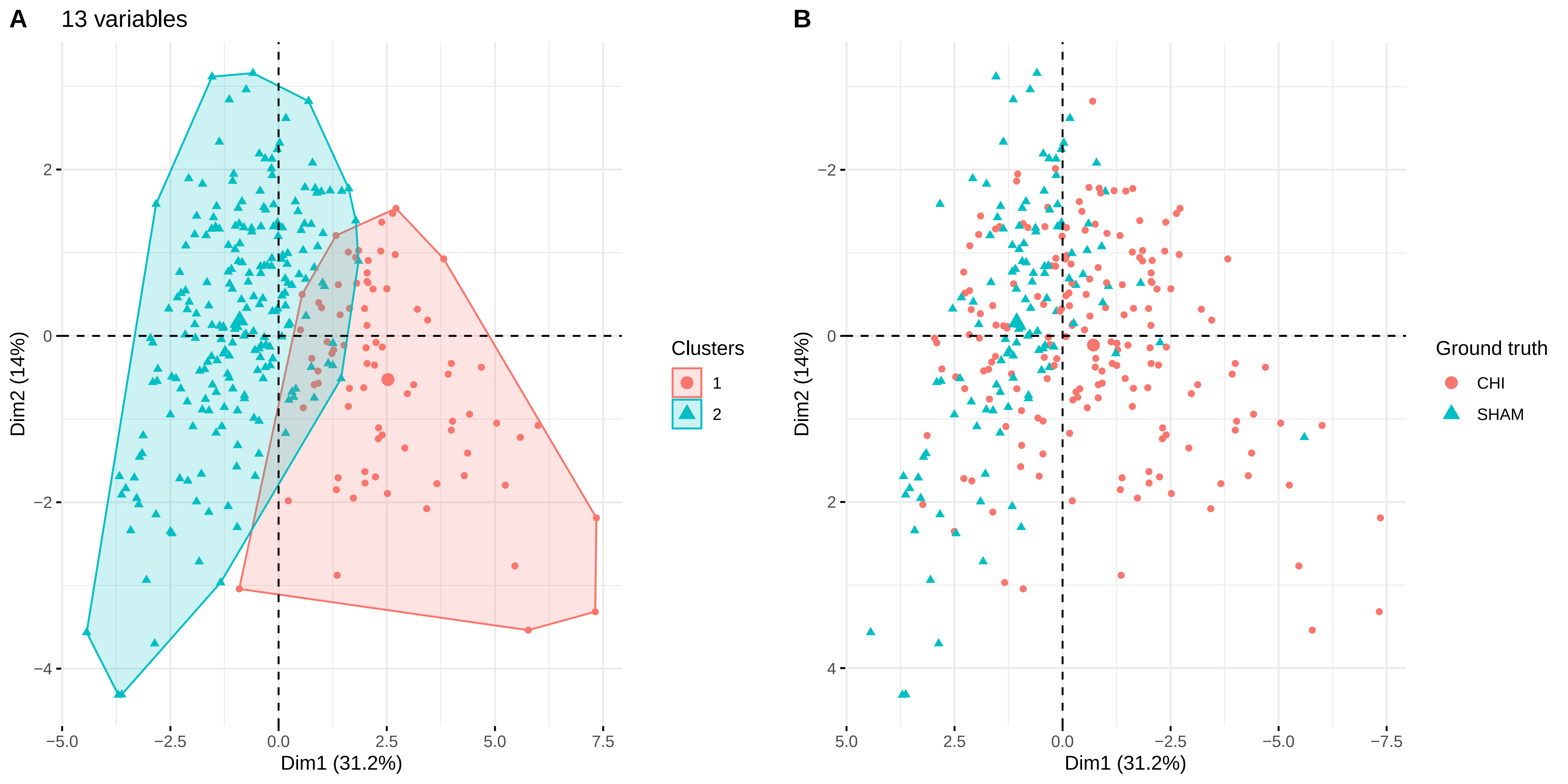
A
13 variables
B
Dim1 (31.2%)
Dim2 (14%)
Clusters
1
2
Ground truth
CHI
SHAM
−5.0
−2.5
0.0
2.5
5.0
7.5
2
0
−2
−4

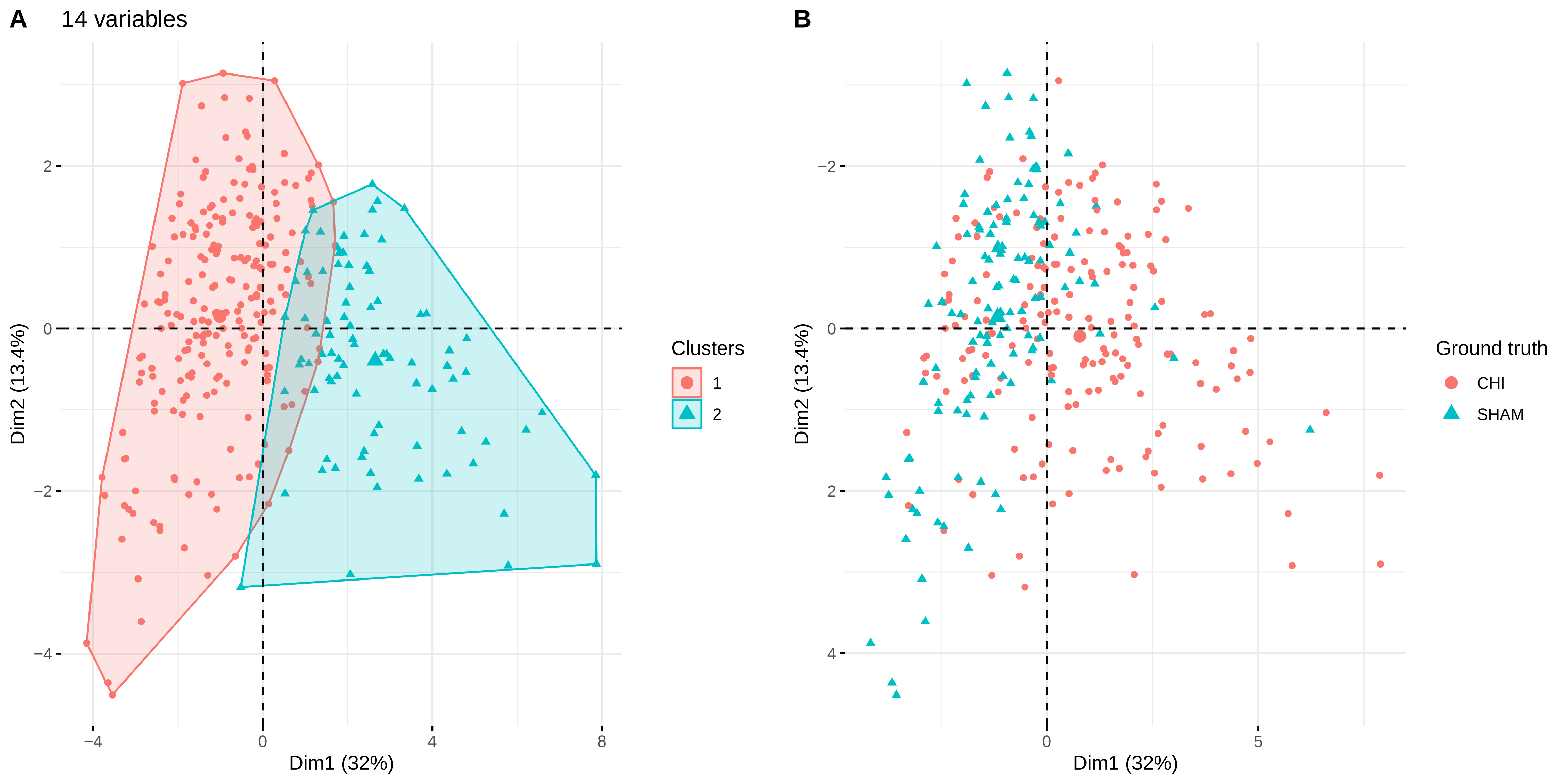

A
14 variables
B
Dim1 (32%)
Dim2 (13.4%)
Clusters
1
2
Ground truth
CHI
SHAM

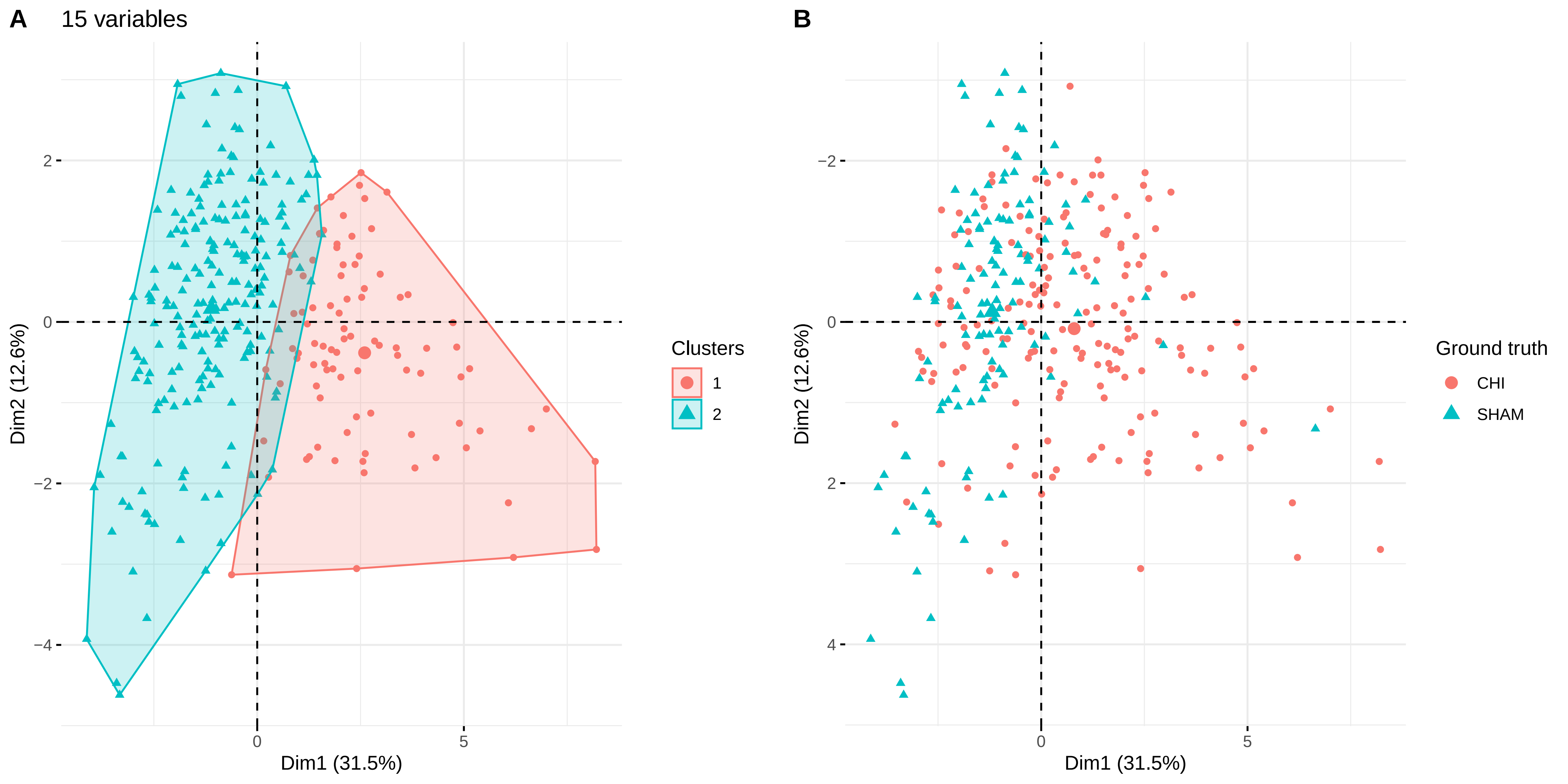
A
15 variables
Dim1 (31.5%)
Dim2 (12.6%)
Clusters
1
2
B
Dim1 (31.5%)
Dim2 (12.6%)
Ground truth
CHI
SHAM
